# T cell receptor–engineered T cells targeting the TP53^R248Q^ neoantigen elicit antitumor effects in human cancer models

**DOI:** 10.1172/JCI196613

**Published:** 2026-01-13

**Authors:** Lianghua Shen, Ziyu Chen, Jian Xu, Qiaomei He, Changmeng Zhang, Xiao Zhou, Xiaodan Ding, Jinan Fang, Fanlin Li, Ming Jiao, Yuqin Yang, Baoxia Dong, Liping Wan, Xueying Ding, Yan Zheng, Jingyi Zhou, Chijian Zuo, Tian Min, Ming Zhu, Bin Ma, Yuhua Wan, Qiufang Guo, Hua Zhang, Jian Hua, Pengran Wang, Qi Li, Jiang Long, Xianmin Song, Yan Zhang

**Affiliations:** 1 Department of Hematology, Shanghai General Hospital, Shanghai Jiao Tong University School of Medicine, Shanghai, China.; 2 Engineering Technology Research Center of Cell Therapy and Clinical Translation, Science and Technology Committee of Shanghai Municipality, Shanghai, China.; 3 Department of Pancreatic Surgery, Shanghai General Hospital, and; 4 Cancer Center, Shanghai General Hospital, Shanghai Jiao Tong University School of Medicine, Shanghai, China.; 5 Suzhou CureMed Biopharma Technology Co. Ltd., Suzhou, China.; 6 KuaiXu Biotechnologies Co. Ltd., Shanghai, China.; 7 SPH Biotherapeutics (Shanghai) Co. Ltd., Shanghai, China.

**Keywords:** Immunology, Oncology, Cancer immunotherapy, p53

## Abstract

Malignant tumors with TP53 mutations exhibit poor therapeutic outcomes and high recurrence rates. T cell receptor–based (TCR-based) T cell therapy shows great promise for targeting intracellular cancer neoantigens. However, the immunogenic potential of TP53 hotspot mutations remains poorly characterized. Here, we identified an immunogenic neoantigen derived from the recurrent TP53^R248Q^ mutation, presented by the prevalent HLA-A*11:01 allele. Additionally, we isolated a TP53^R248Q^-reactive TCR that specifically recognized the TP53^R248Q^ mutation without any discernible cross-activity with cognate WT TP53 or other TP53 mutants at the same codon position. Functional characterization revealed that TP53^R248Q^ TCR-T cells exhibited selective cytotoxicity against tumor cells expressing both the TP53^R248Q^ mutation and HLA-A*11:01 in vitro. Importantly, the adoptive transfer of TP53^R248Q^ TCR-T cells exhibited significant antitumor activity in a clinically relevant patient-derived xenograft model engrafted with TP53^R248Q^/HLA-A*11:01–positive human tumor tissues. Collectively, our study validates the immunogenicity of the TP53^R248Q^ hotspot mutation and provides a TCR with high therapeutic potential for the development of T cell therapies targeting TP53^R248Q^/HLA-A*11:01–positive cancers.

## Abstract 2

A novel TCR-T cell therapy against cancers harboring the recurrent TP53^R248Q^ hotspot mutation

## Introduction

TP53 is the most frequently mutated tumor suppressor gene in over 50% of human cancers (1, 2). TP53 mutations confer substantial growth advantages to cancer cells and correlate strongly with therapeutic resistance and poor prognoses. Approximately 90% of TP53 mutations across virtually all cancer subtypes are missense mutations, predominantly clustered at amino acid sites R175, G245, R248, R249, R273, R282, and Y220 (3, 4). Such substitutions induce either thermodynamic destabilization of the TP53’s tertiary structure or alter DNA-binding specificity, thereby abrogating its tumor-suppressive functions through either structural destabilization or functional inactivation of the DNA-binding domains. Despite decades of intensive drug discovery efforts targeting mutant TP53, including pharmacological reactivators designed to restore wild-type conformation and transcriptional activity, no mutant TP53-targeted therapeutic has achieved FDA approval to date (5, 6).

T cell receptor–engineered T (TCR-T) cell therapy represents an immunotherapeutic approach wherein autologous T cells were genetically engineered to express tumor antigen-specific TCRs, enabling precise recognition and elimination of malignant cells (7). This strategy capitalizes on tumor-specific neoantigens — aberrant peptides generated from somatic mutations that are exclusively presented on cancer cells via HLA molecules — thereby providing ideal therapeutic targets with minimal on-target/off-tumor toxicity (8). However, not all mutation gives rise to neoantigen. Only those mutations that generate altered peptides capable of being efficiently processed and presented by HLA molecules and, subsequently, activate T lymphocytes can be termed as neoantigens. In contrast, patient-specific or private neoantigens predominantly arise from random passenger mutations and public or shared neoantigens arise from recurrently mutated driver genes, such as TP53, KRAS, and PIK3CA mutations, among others (9–11). These public neoantigens are clonally conserved and shared across patients and cancer types, making them highly attractive targets for TCR-T–based immunotherapies (12, 13). Notably, a recent clinical study demonstrated that a patient with refractory breast cancer achieved objective tumor regression following treatment with TCR-T cells targeting the HLA-A*02:01–restricted TP53^R175H^ mutation (9). This evidence further underscores the potential of TCR-based immunotherapy as a viable alternative for treating cancers harboring TP53 mutations. Although neoantigens derived from the TP53^R175H^ and TP53^Y220C^ hotspot mutations have been identified in the context of HLA-A*02:01 allele (14), which is one of the most prevalent alleles in the White population (15), the immunogenicity of the remaining TP53 mutations remains largely underexplored. Therefore, identification of neoantigens derived from other TP53 mutations and discovery of their responsive TCRs are highly desirable.

In the present study, we successfully identified a 9–amino acid neopeptide (SCMGGMNQR) derived from the TP53^R248Q^ mutation. This mutation is among the most common TP53 mutations in both hematological malignancies and solid tumors. The identified neopeptide is capable of being processed and presented by the HLA-A*11:01 allele, which is the most prevalent HLA allele in East Asian populations, especially Chinese populations (16, 17). Furthermore, we isolated a TCR that specifically targets the TP53^R248Q^ mutation from HLA-A*11:01–positive healthy donors (HDs). In both in vitro and in vivo settings, TP53^R248Q^ TCR-T cells specifically and efficiently killed tumor cell lines as well as primary human cancer tissues that harbored both HLA-A*11:01 and TP53^R248Q^ mutations. Collectively, our findings have uncovered a public TP53 neoantigen and provided a promising TCR with therapeutic potential for patients with cancer who are positive for both the HLA-A*11:01 allele and the TP53^R248Q^ mutation.

## Results

### Identification and characterization of a neopeptide derived from TP53^R248Q^ mutation.

**Figure 1:**
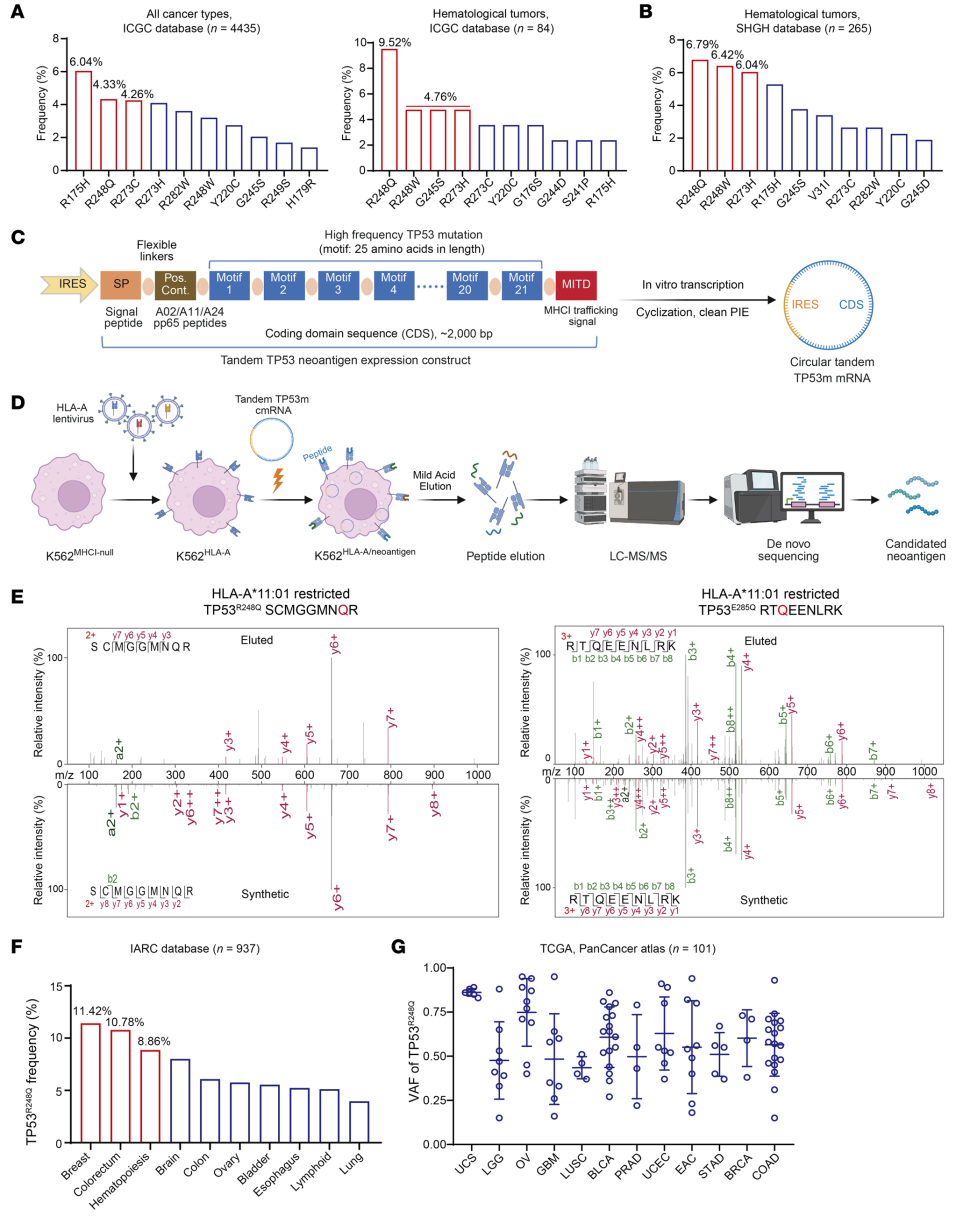
Identification of TP53 antigenic peptides and their HLA restriction. (**A**) ICGC database analysis of top 10 TP53 site mutation and frequency information in patients with all cancer types (*n* = 4435) or hematological tumors (*n* = 84) with concomitant TP53 mutations. (**B**) Clinical data from 265 patients at Shanghai General Hospital with hematologic malignancy with TP53 site mutations. (**C**) Vector map of the tandem TP53 neoantigen expression constructs used in this study. (**D**) Process overview for the K562 cell engineering and MAE/MS analysis of neopeptides presented by tandem TP53 mutation–expressing HLA monoallelic K562 cell lines. (**E**) TP53^R248Q^ neopeptide (SCMGGMNQR) and TP53^E285Q^ neopeptide (RTQEENLRK) were found to be specifically presented by HLA-A*11:01 through MAE/MS. (**F**) Comprehensive analysis of the IARC TP53 database revealed the TP53^R248Q^ hotspot mutation frequency exhibited in different cancer types. (**G**) Analysis of The Cancer Genome Atlas (TCGA) database for TP53^R248Q^-positive pan-cancer patient samples shows high VAF for TP53^R248Q^ in a large fraction of patients (*n* = 101).

Analysis of the International Agency for Research on Cancer (IARC) TP53 database (https://www.iarc.who.int/) revealed that patients carrying TP53 mutations — whether with all analyzed cancer types (Supplemental Figure 1A; supplemental material available online with this article; https://doi.org/10.1172/JCI196613DS1) or hematologic malignancies (Supplemental Figure 1B) — exhibited significantly lower survival rates compared with those with TP53 WT. Pan-cancer analysis of 4,435 TP53 mutations in the International Cancer Genome Consortium (ICGC) database (https://platform.icgc-argo.org/) revealed the top 10 recurrent TP53 missense mutations (Figure 1A), with TP53^R248Q^ ranking first in hematologic malignancies (9.52% prevalence, *n* = 84) and second in all cancer types (4.33% prevalence, *n* = 4,435). We also analyzed clinical data from 265 patients with hematologic malignancy with TP53 mutations at Shanghai General Hospital, which revealed that the TP53^R248Q^ mutation demonstrated the highest frequency (6.79%) among all detected variants (Figure 1B). We have recently demonstrated a strategy that combined circular mRNA–mediated (cmRNA-mediated) target protein expression with mild acid elution/mass spectrometry–based (MAE/MS-based) de novo peptide sequencing for the identification of HLA-bound immunopeptides (18). By using this strategy, we synthesized a cmRNA vector encoding a polypeptide composed of tandem TP53 hotspot mutations at positions R175, Y220, G245, R248, R273, R282, and E285 (Supplemental Table 1). To prevent splicing across TP53 mutation modules, spacers were inserted between different modules (19), a signal peptide was inserted at the start of the vector, and a MHC-I trafficking signal domain element was added at the end (Figure 1C) (20). cmRNA was synthesized via T7 RNA polymerase–mediated in vitro transcription, with subsequent circularization employing the permuted intron-exon methodology (21). This integrated approach effectively circumvents the requirement for conventional mRNA processing steps, including 5′ capping, 3′ polyadenylation, and nucleoside base modification. The mutant TP53 cmRNAs were then electrotransferred into the K562 cell line, which was engineered to ectopically overexpress the individual HLA-A*11:01, HLA-A*02:01, or HLA-A*24:02 alleles. HLA-bound peptides were retrieved via MAE/MS analysis. Protein profiling was employed to identify peptides through de novo sequencing (Figure 1D). The average lengths of the target peptides obtained by MAE/MS primary ranged from 8 to 11 amino acids, which was consistent with the HLA-I class presentation pattern (Supplemental Figure 1C). Notably, a TP53^R248Q^ neopeptide (SCMGGMNQR) and a TP53^E285Q^ neopeptide (RTQEENLRK) were found to be specifically presented by HLA-A*11:01 (Figure 1E and Supplemental Figure 1D). The sequences of the eluted TP53^R248Q^ and TP53^E285Q^ neopeptides were further corroborated by comparing their mass spectra with those of synthetic peptides (Figure 1E). We also conducted in silico prediction for HLA-A*11:01–restricted high-affinity TP53 mutant candidate peptides using NetMHCpan4.1. Among the 9 predicted candidate peptides with strong or weak binding affinity, only 1 peptide (TP53^E285Q^, RTQEENLRK) was verified in the MS assay (Supplemental Figure 1E).

Intriguingly, although TP53^R248Q^ (SCMGGMNQR) peptide was identified in the MAE/MS assay, it was absent from the prediction list of HLA-A*11:01–binding peptides generated by NetMHCpan4.1. This suggests that the MS-based approach can complement in silico prediction tools for the precise identification of neopeptides. To further validate the immunogenicity of this neoantigen, we performed MAE/MS using clinically relevant pancreatic ductal adenocarcinoma (PDAC) patient tissue (HLA-A*11:01^+^/TP53^R248Q+^, Supplemental Table 2). Then, we successfully identified the TP53^R248Q^ neopeptide (SCMGGMNQR) in PDAC patient tissue, and its sequence matched the TP53^R248Q^ antigen peptide presented by K562 cell lines overexpressing the HLA-A*11:01 allele (K562^A11^) (Supplemental Figure 1F). To experimentally validate the binding between the TP53^R248Q^ neopeptide and HLA-A*11:01, we performed pMHC complex stability assays. The results demonstrated that the TP53^R248Q^ neopeptide-HLA-A*11:01 complex exhibited a half-life (*t*_1/2_) of 10.1 hours, which is much longer than that of the TP53^R248WT^ peptide-HLA-A*11:01 (*t*_1/2_ = 1.8 hours), indicating markedly stronger binding of the TP53^R248Q^ neopeptide to HLA-A*11:01 (Supplemental Figure 1G).

Comprehensive analysis of the IARC TP53 database also revealed that the TP53^R248Q^ hotspot mutation exhibits high frequencies in breast cancer, colorectal cancer, and hematologic malignancies (Figure 1F). Then, we examined the variant allele frequency (VAF) within an extensive pan-cancer cohort consisting of *n* = 101 patient samples harboring the TP53^R248Q^ mutation. Our findings revealed that TP53^R248Q^ mutation was clonally expressed across a spectrum of diverse tumors, with a mean VAF of 58.6%. In the case of uterine carcinosarcoma (*n* = 6), the mean VAF reached as high as 86% (Figure 1G). Taking into account that the TP53^R248Q^ mutation ranked as the most common TP53 mutations in hematological malignancies, and the second-most common in solid tumors, and also considering that HLA-A*11:01 ranked as the most prevalent HLA type in the East Asian population, we thus decided to choose the TP53^R248Q^ (SCMGGMNQR) neopeptide for further investigation.

### TP53^R248Q^ neopeptide immunogenicity and isolation of TP53^R248Q^-reactive TCR.

**Figure 2:**
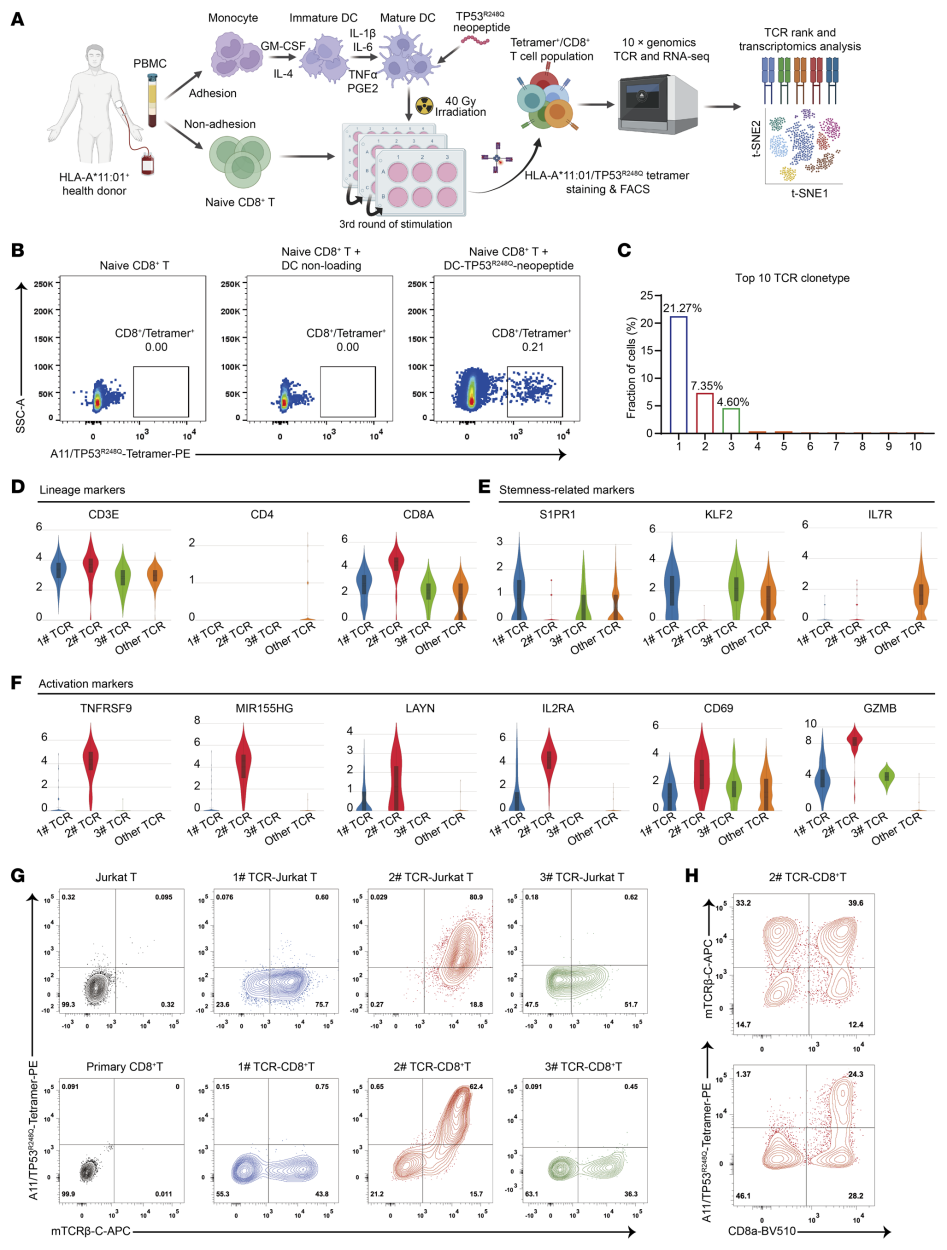
Identification of HLA-A*11:01–restricted TP53^R248Q^-responsive TCR by tetramer staining combined with single-cell TCR sequencing. (**A**) Schematic diagram of in vitro stimulation of T cells specific to TP53^R248Q^, followed by isolation TP53^R248Q^ TCRs by tetramer staining combined with single-cell TCR sequencing. (**B**) After the third round of in vitro stimulation, CD8^+^/HLA-A*11:01–TP53^R248Q^ tetramer^+^ T cells were sorted using flow cytometry. (**C**) The frequency of top 10 TCR clonotypes was ranked after single-cell TCR sequencing. (**D**–**F**) Single-cell transcriptomics analyses the expression of lineage-related, stemness-related, and activation-related markers between the top 3 T cell clones and other T cell clones. (**G**) In vitro binding assay validation by HLA-A*11:01–TP53^R248Q^ tetramer staining after lentiviral infection with TP53^R248Q^ TCR clones 1, 2, and 3. (**H**) Flow cytometry identification of the CD8 coreceptor–dependent manner of TP53^R248Q^ TCR clone 2.

We explored whether the HLA-A*11:01–presented TP53^R248Q^ neopeptide could elicit T cell recognition. To this end, HLA-A*11:01–restricted TP53^R248Q^ tetramers were generated for subsequent TCR screening (Supplemental Figure 2, A and B). Given that several prior studies have demonstrated the identification of neoantigen-specific TCRs from HDs, we pulsed monocyte-derived dendritic cells (moDCs) obtained from HLA-A*11:01–positive HDs (*n* = 8, Supplemental Table 3) with the TP53^R248Q^ neopeptide (SCMGGMNQR) and cocultured them with autologous naive CD8^+^ T cells from the same donor (Figure 2A). Following 3 rounds of in vitro stimulation with neopeptide-loaded moDCs, flow cytometry analysis detected a small but distinct tetramer-positive CD8^+^ T cell proportion (~0.21%) in 1 donor (Figure 2B). The CD8^+^tetramer^+^ double-positive T cells were FACS sorted for single-cell TCR and transcriptome sequencing. Three dominant clones accounting for 21.27%, 7.35%, and 4.6% of cells were identified based on unique TCR sequences and clonal expansion (Figure 2C and Supplemental Figure 2C). All clones exhibited a CD3e^+^CD8a^+^CD4^–^ phenotype (Figure 2D), confirming the HLA class I restriction. Single-cell transcriptomic profiling revealed that T cell activation markers (such as TNFRSF9, MIR155HG, IL-2RA, LAYN, GzmB, etc.) were marked upregulation, while stemness-associated genes (S1PR1, KLF2, and IL7R) were substantially downregulated in clone 2 (Figure 2, E and F), suggesting clone 2 underwent preferential expansion upon TP53^R248Q^ neopeptide stimulation (Supplemental Figure 2D).

To further test whether these candidate TCRs truly recognize the TP53^R248Q^ neopeptide presented by HLA-A*11:01, Jurkat T cells or PBMCs from third-party HDs were lentivirally transduced with codon-optimized TCR genes, and mouse TCR consistent regions were used to minimize mispairing between introduced and endogenous TCR (Supplemental Figure 2E). Using the HLA-A*11:01–restricted TP53^R248Q^ tetramer staining and flow cytometry analysis, a clear tetramer^+^mTCRβC^+^ population appeared in Jurkat T or primary T cells transfected with clone 2 TCR but not clone 1 or 3 TCRs (Figure 2G). Thus, consistent with the transcriptomic characterization results, the tetramer staining indicated that the clone 2 TCR was a bona fide responsive TCR that specifically recognized the TP53R248Q neopeptide. Additionally, only CD8^+^ T cells but not CD4^+^ T cells that transduced with TP53^R248Q^-reactive TCR were preferably stained with tetramer, indicating that the TCR recognize TP53^R248Q^ neopeptide presented by HLA-A*11:01 in a CD8 coreceptor–dependent manner (Figure 2H). Collectively, our results showed that TP53^R248Q^ neopeptide (SCMGGMNQR) is truly immunogenic and can be presented by HLA-A*11:01 and specifically recognized by a reactive TCR from HLA-A*11:01–positive HDs T cells.

### Validation and functional characterization of TP53^R248Q^ TCR-T cells.

**Figure 3:**
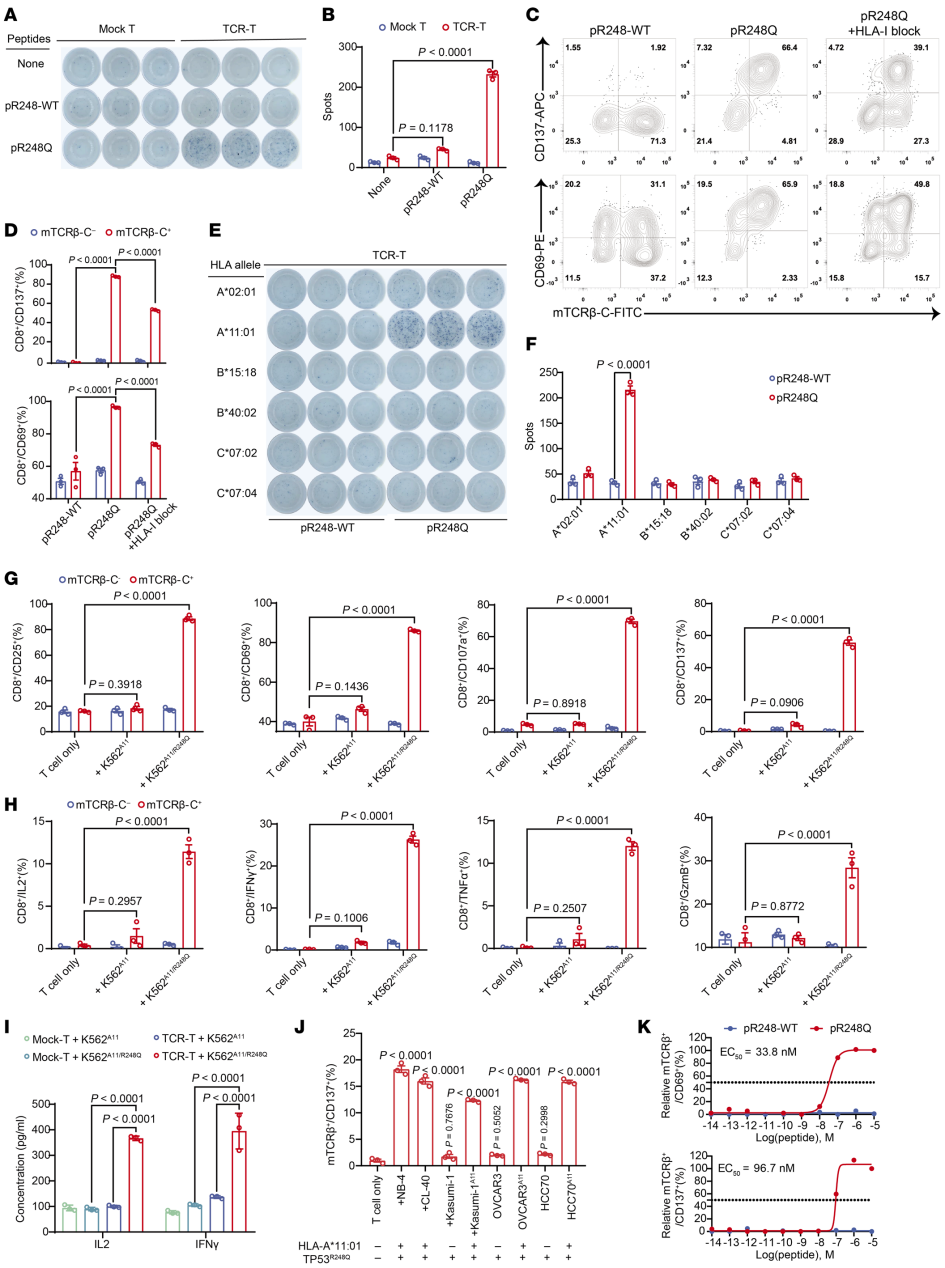
TP53^R248Q^ TCR-T cells specifically recognize cognate neopeptide presented by HLA-A*11:01 allele. (**A** and **B**) Representative IFN-γ ELISpot images and quantitative analysis of TP53^R248Q^ TCR-T cell activation. Mock-T or TCR-T cells were cocultured for 24 hours with moDCs pulsed with TP53^R248Q^ neopeptide (pR248Q, 5 μg/mL) or TP53^R248WT^ peptide (pR248-WT, 5 μg/mL) or were unpulsed. (**C** and **D**) TCR-T cells were cocultured with antigenic peptide-pulsed moDCs and HLA-I blocking antibody (W6/32, 1 μg/mL) for 24 hours. Subsequently, the expression of T cell activation markers (CD137 and CD69) was analyzed and quantified using flow cytometry. (**E** and **F**) TCR-T cells were cocultured for 24 hours with monoallelic K562 pulsed with TP53^R248Q^ neopeptide or TP53^R248WT^ peptide. (**G** and **H**) Differences in the expression levels of activation-associated markers and cytokines in TCR-T cells cocultured with control and target cells for 24 hours were detected using flow cytometry. (**I**) Mock-T or TCR-T cells were cocultured with control and target cells for 24 hours. Changes in the secretion levels of IL-2 and IFN-γ cytokines in the supernatant of the coculture system were detected using ELISA. (**J**) The CD137 expression level of TCR-T cells was detected by flow cytometry after coculturing with different tumor cells for 24 hours. (**K**) 10^–14^ M to 10^–5^ M of the TP53^R248Q^ neopeptide or TP53^R248WT^ peptide was loaded into K562^A11^ cells, which were subsequently cocultured for 24 hours with TCR-T cells, respectively. Changes in the expression levels of CD137 and CD69 in the TCR-T cells were detected by flow cytometry, and the EC_50_ values of TCR-T cells were calculated. Data are presented as the mean ± SEM; *n* = 3. In **B**, **D**, and **F**–**I**, 2-way ANOVA were used. In **J**, a 1-way ANOVA was used.

We next examined whether the TP53^R248Q^ neopeptide could specifically activate TP53^R248Q^-responsive TCR-T cells in an HLA-A*11:01–restricted manner. Using an IFN-γ-ELISpot assay, we confirmed that TP53^R248Q^ TCR-T cells were activated exclusively in response to moDCs loaded with TP53^R248Q^ neopeptide but not to those loaded with the WT TP53 peptide (Figure 3, A and B). To further examine whether the TP53^R248Q^-responsive TCR was HLA-I restricted, an HLA-I–blocking antibody (W6/32) was added to a coculture system containing TP53^R248Q^ TCR-T cells and neopeptide-pulsed moDCs. The activation of TCR-T cells was measured by flow cytometry. The expression of CD137 and CD69, which are surface markers associated with T cell activation, was specifically attenuated on TP53^R248Q^ TCR-T cells upon the addition of the HLA-I–blocking antibody, indicating HLA-I restriction (Figure 3, C and D). To further confirm HLA-A*11:01 allele dependency, we constructed monoallelic K562 cell lines expressing individual HLA-A/B/C alleles from the same donor from which the TP53^R248Q^ TCR was derived. Coculture of these TP53^R248Q^ neopeptide-pulsed monoallelic K562 cell lines with TCR-T cells revealed INF-γ secretion exclusively in the context of HLA-A*11:01 allele. Thus, these results demonstrate that TP53^R248Q^ TCR-T cells specifically recognize the TP53^R248Q^ neopeptide presented by HLA-A*11:01 allele, while displaying no cross-reactivity with other HLA alleles from the same donor (Figure 3, E and F).

To further assess whether endogenously processed TP53^R248Q^ mutant protein-derived neopeptides could be presented and recognized by TP53^R248Q^ TCR-T cells, we generated K562-based target cell lines by overexpressing both the HLA-A*11:01 allele and the TP53^R248Q^ mutant gene. We observed that the K562-HLA-A*11:01–TP53^R248Q^ (K562^A11/R248Q^) cells specifically induced upregulation of activation markers (CD25, CD69, CD107a, and CD137) on TP53^R248Q^ TCR-T cells (mTCRβ positive), in contrast to T cells that were not transfected with TP53^R248Q^ TCR (mTCRβ negative) (Figure 3G). Next, we assessed the secretion of cytokines and the release of cytotoxic molecules in TP53^R248Q^ TCR-T cells following coculture with K562^A11/R248Q^ target cells using flow cytometry. As shown in Figure 3H, T cells transduced with TP53^R248Q^ TCR efficiently secreted cytokines (IFN-γ, TNF-α, and IL-2) and released granzyme B (GzmB) upon coculture with target cells. ELISA assays further confirmed the specificity of TCR-T cell recognition, demonstrating significant upregulation of IL-2 and IFN-γ cytokine secretion levels when targeting K562^A11/R248Q^ cells (Figure 3I).

Similarly, NB4 (acute promyelocytic leukemia cells) and CL40 (colon adenocarcinoma cells), which both harbor the HLA-A*11:01 allele and the TP53^R248Q^ mutation, potently activated TP53^R248Q^ TCR-T cells, as evidenced by CD137 upregulation (Figure 3J). These results demonstrated that endogenously processed TP53^R248Q^ neopeptides are naturally presented by HLA-A*11:01 on tumor cells and efficiently activate TP53^R248Q^ TCR-T cells. Notably, following HLA-A*11:01 overexpression in Kasumi-1, OVCAR3, and HCC70 cells (HLA-A*11:01–negative/TP53^R248Q^-positive), TP53^R248Q^ TCR-T cells cocultured with these cells exhibited significantly upregulated CD137 (Figure 3J) and CD69 expression levels (Supplemental Figure 3, A and B). Consistent with these findings, K562^A11/R248Q^ and NB4 cells, in contrast to K562^A11^ control cells, specifically activated a Jurkat T-NFAT-luciferase reporter cell line expressing TP53^R248Q^ TCR in vitro (Supplemental Figure 3, C and D).

Ultimately, we evaluated the sensitivity of TP53^R248Q^-responsive TCR to its cognate neopeptide by determining its functional avidity. TP53^R248Q^ TCR-T cells were cocultured with K562^A11^ pulsed with titrated concentrations of either the TP53^R248Q^ neopeptide or the corresponding WT peptide. Surface markers associated with T cell activation were then measured by flow cytometry. The half-maximal effective concentrations (EC_50_) determined by CD69 or CD137 expression were approximately 33.8 nM and 96.7 nM, respectively (Figure 3K). In contrast, the TP53^R248Q^ TCR-T cells exhibited no reactivity toward the corresponding WT peptide across all the tested concentrations. Collectively, these findings demonstrate that the TP53^R248Q^ TCR-T cells are efficiently activated in vitro in a manner restricted by HLA-A*11:01–restricted and dependent upon the TP53^R248Q^ mutation.

### TP53^R248Q^ TCR-T cells specifically recognize the TP53^R248Q^ neopeptide/HLA-A*11:01 complex without discernible cross-reactivity.

**Figure 4:**
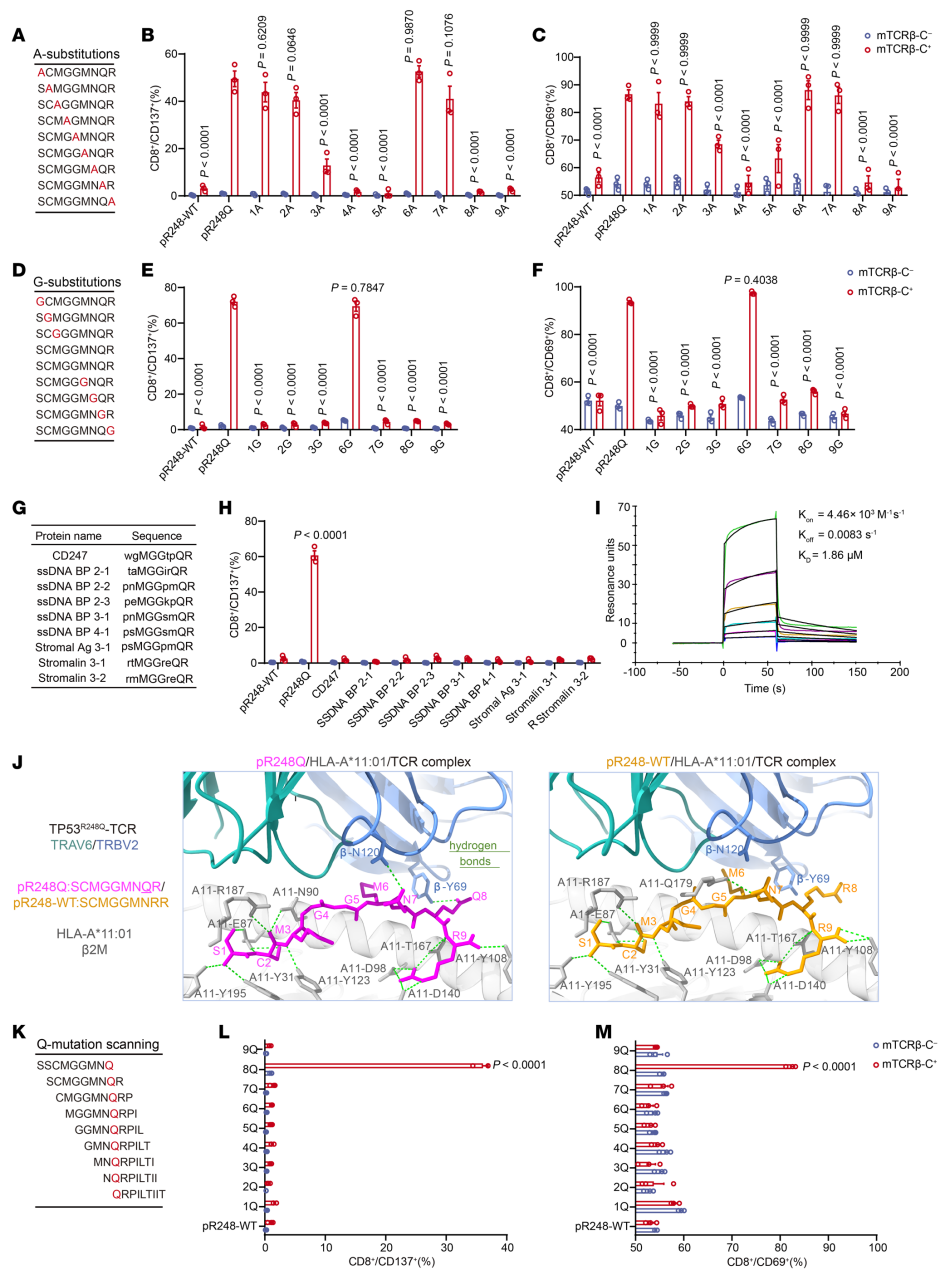
TP53^R248Q^ TCR-T cells recognize TP53^R248Q^ neopeptide/HLA-A*11:01 complex with high specificity and low cross-reactivity profile. (**A**) Schematic representation of the 9-peptide sequence used in the “A” substitution experiments. (**B** and **C**) K562^A11^ cells were loaded with (5 μg/mL) TP53^R248WT^, TP53^R248Q^, and TP53^R248Q^ neopeptides with different “A” substitution sites and then cocultured for 24 hours with TCR-T cells. Subsequently, flow cytometry was performed to detect the expression levels of CD137 and CD69 in TCR-T cells. (**D**) Schematic representation of the 9-peptide sequence used in the “G” substitution experiments. (**E** and **F**) K562^A11^ cells were loaded with (5 μg/mL) TP53^R248WT^, TP53^R248Q^, and TP53^R248Q^ neopeptides with different “G” substitution sites, and then the expression levels of CD137 and CD69 in TCR-T cells was detected. (**G**) Peptides for which the “xxMGGxxQR” motif is present in the human proteome database, and their protein names were retrieved using PROSITE. (**H**) Nine predicted peptides were loaded onto K562^A11^ cells, and then the expression levels of CD137 in TCR-T cells were detected. (**I**) Representative SPR sensorgram measuring the dissociation constant (K_D_) for TP53^R248Q^ TCR to the pR248Q/HLA-A*11:01 complex. The curves show kinetic fits (concentration: 250–8,000 nM). (**J**) Structural overview of the TP53^R248Q^ TCR-pR248Q/HLA-A*11:01 or TP53^R248Q^ TCR-pR248WT/HLA-A*11:01 ternary complex. (**K**) Schematic representation of the 9-peptide sequence used in the “Q” mutation scanning experiments. (**L** and **M**) K562^A11^ cells were loaded with (5 μg/mL) TP53^R248WT^ or TP53^R248Q^ neopeptides with different “Q” mutation sites, and then the expression levels of CD137 and CD69 in TCR-T cells (cocultured for 24 hours) was detected. Data are presented as the mean ± SEM; *n* = 3. In **B**, **C**, **E**, **F**, **H**, **L**, and **M**, 2-way ANOVA were used. The *P* values for **B**, **C**, **E**, and **F** were relative to the TP53^R248Q^ neopeptide group, and the *P* values for **H**, **L**, and **M** were relative to the TP53^R248WT^ peptide group.

Given that undesirable TCR cross-reactivity can lead to severe off-target toxicity, it is critical to carefully evaluate this during the preclinical stage. To characterize the recognition motif and specificity of TP53^R248Q^ TCR-T cells, alanine (A) and glycine (G) substitution assays were performed, wherein each residue of the TP53^R248Q^ neopeptides was sequentially substituted with alanine or glycine. TP53^R248Q^ TCR-T cells were cocultured with K562^A11^ cells pulsed with the substituted peptides, and the effect of these substitutions on TP53^R248Q^ TCR-T cell activation was analyzed by flow cytometry. Notably, alanine substitutions at positions 3, 4, 5, 8, and 9 resulted in significant reduction in CD137 and CD69 upregulation compared with stimulation with the unmodified TP53^R248Q^ neopeptide, indicating the core recognition motif “xxMGGxxQR” (Figure 4, A–C). Furthermore, glycine substitutions at positions 1, 2, 3, 7, 8, and 9 resulted in an approximately 90% reduction in both CD137 and CD69 expression on TP53^R248Q^ TCR-T cells (Figure 4, D–F). These results highlight the essential role of the glutamine (Q) residue at position 8 in mediating recognition by the TP53^R248Q^-reactive TCR.

To investigate whether any self-antigens in the human proteome might be cross-recognized by the TP53^R248Q^ TCR, we queried the “PROSITE” database. We identified a total 9 peptide fragments derived from 4 proteins (CD247, ssDNA binding protein, stromalin-3, and stromal antigen 3) that matched the “xxMGGxxQR” motif (Figure 4G). All 9 predicted peptides were then synthesized and loaded onto K562^A11^ cells, and none of them active TP53^R248Q^ TCR-T cells (Figure 4H and Supplemental Figure 4A). These results suggest that there is no or limited evidence of cross-reactivity of TP53^R248Q^ TCR-T cells against the predicted peptides encoded in the human proteome. Then, we expressed and purified the TP53^R248Q^-specific TCR protein (Supplemental Figure 4, B and C) and quantitatively measured its binding affinity toward the TP53^R248Q^ neopeptide-HLA-A*11:01 complex using surface plasmon resonance (SPR). The results demonstrated an affinity constant (K_D_) of 1.86 μM for TP53^R248Q^-TCR (Figure 4I). This measured affinity aligns well with the functional potency observed in our cellular assays and falls within the range for clinically relevant, tumor neoantigen-specific TCRs (22–24), thereby supporting its potential as a therapeutic candidate.

To further investigate the specificity of the TP53^R248Q^ TCR at the molecular level, we predicted the atomic structure of the TP53^R248Q^ neopeptide/HLA-A*11:01/TCR complex. Utilizing Alphafold3, one of the most advanced protein structure prediction tools, we generated a molecular model with a high interface confident score. Our analysis revealed that the TP53^R248Q^ neopeptide adopts a backbone conformation similar to those of the previously reported TP53^R175H^ and KRAS^G12V^ neopeptides (9, 25) (Supplemental Figure 4D). Subsequently, we conducted a ChimeraX analysis of the interaction, including hydrogen bonds, between the TP53^R248Q^ neopeptide and its surrounding residues (Figure 4J). Our findings indicate that the Ser 1, Cys 2, Met 3, and Arg 9 residues of the TP53^R248Q^ neopeptide were deeply embedded within the binding pocket of the HLA-A*11:01 heavy chain, forming exclusively with the HLA-A*11:01 heavy chain. This suggest that these terminal residues play a crucial role in anchoring the peptide. Disruption of these residues would likely destabilize the presentation of the TP53^R248Q^ neopeptide by HLA-A*11:01, resulting in impaired TCR recognition and T cell activation (Figure 4J and Supplemental Figure 4E). The central residues of TP53^R248Q^ neopeptides primarily interact with the complementarity-determining regions (CDRs) of the TCRα and -β chains. Notably, Asn 7 and Gln 8 form hydrogen bonds with Asn 120 (CDR3 of the TCRβ chain) and Tyr 69, respectively (Supplemental Figure 4, E and F). Additionally, we predicted the structure of the TP53^R248WT^ peptide/HLA-A*11:01/TCR (Figure 4J). In this model, we observed the loss of hydrogen bonds between the TP53^R248WT^ peptide and the TCRα and β chains when the P8 residue was Arg, underscoring the critical role of P8 Gln in TCR specificity. These findings explain why our TCR cannot recognize the TP53^R248WT^ peptide (Supplemental Figure 4, E–G). Combined with our A/G substitution assay, these structural analyses provide a molecular basis for the specificity and low cross-reactivity of our TP53^R248Q^ TCR.

Next, we examined the mutated amino acid glutamine (Q) position of the antigenic peptide “SCMGGMNQR” in an in vitro activation assay (Figure 4K), and the flow cytometry results showed that only the peptide of the antigenic peptide “SCMGGMNQR” could specifically activate TP53^R248Q^ TCR-T cells and upregulate CD137 and CD69 expression (Figure 4, L and M).

In addition to the TP53^248Q^ mutation, there are other missense mutations at the same position (TP53^R248W^ and TP53^R248L^). We therefore tested the recognition potential of TP53^R248Q^ TCR-T cells toward these mutations. As shown in Supplemental Figure 4H, the results demonstrated that TP53^R248Q^ TCR-T cells specifically recognized the TP53^R248Q^ neopeptide but did not react with other mutations at the R248 position. Collectively, our findings indicate that TP53^R248Q^ TCR-T cells recognize TP53^R248Q^ neopeptide bound to HLA-A*11:01 with relatively high specificity and a low cross-reactivity profile.

### TP53^R248Q^ TCR-T cells efficiently eliminate tumor cells harboring HLA-A*11:01 and TP53^R248Q^ mutation in vitro.

**Figure 5:**
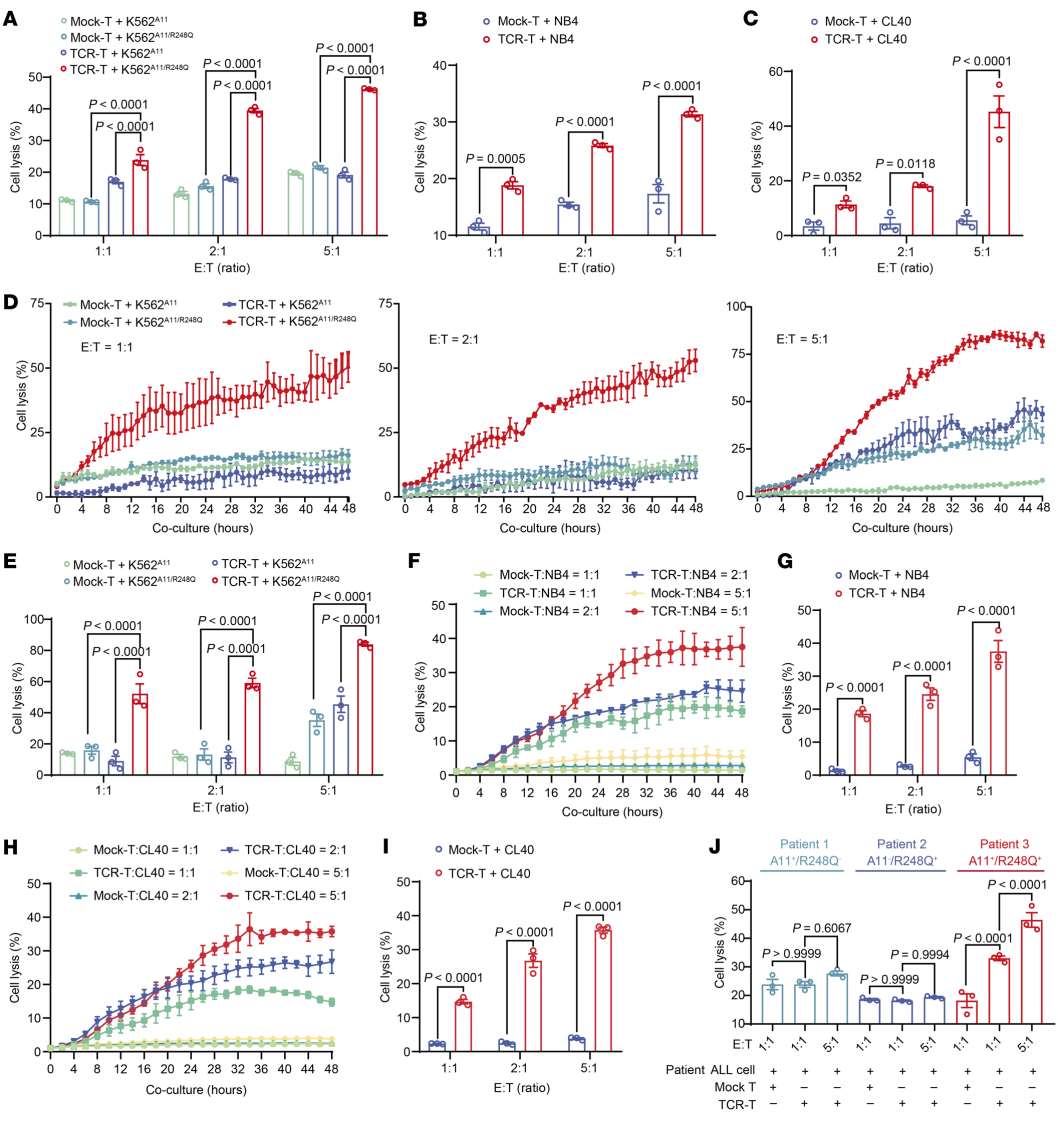
TP53^R248Q^ TCR-T cells specifically recognized and killed TP53^R248Q^/HLA-A*11:01–bearing tumor cells in vitro. (**A** and **B**) Flow cytometry analysis of K562^A11^, K562^A11/R248Q^, or NB4 cell lysis (%) after cocultured for 24 hours with mock-T or TP53^R248Q^ TCR-T cells. Target cell lysis was assessed via CellTrace violet^+^ and LIVE/DEAD^+^ staining. (**C**) The in vitro killing effect of mock-T or TP53^R248Q^ TCR-T cells on CL40 cells was assayed by LDH assay (coculture for 24 hours, E:T = 1:1/2:1/5:1). (**D**) Incucyte live-cell detection of K562^A11^ control cell or K562^A11/R248Q^ target cell lysis (%) at varying E:T ratios over 48 hours. (**E**) Target cell lysis at 48 hours after coculture, analyzed via Incucyte Analysis software. Real time cytotoxicity of TCR-T cells across NB4 (**F** and **G**) and CL40 (**H** and **I**) cells at varying E:T ratios within 48 hours by Incucyte detection. (**J**) Patient-derived tumor cell lysis was assessed via CellTrace violet^+^ and LIVE/DEAD^+^ staining. Data are presented as the mean ± SEM; *n* = 3. In **A**–**C**, **E**, **G**, and **I**, 2-way ANOVA were used. In **J**, a 1-way ANOVA was used.

To characterize the cytotoxic specificity and efficacy of TP53^R248Q^ TCR-T cells, we evaluated their killing activity against K562^A11/R248Q^, NB4, and CL40 cells at various effector-to-target (E:T) ratios. We first analyzed HLA-I expression levels in the aforementioned cell lines by flow cytometry, and the results revealed that NB4, CL40, and K562^A11/R248Q^ cells exhibited relatively low, medium, and high HLA-I expression levels, respectively (Supplemental Figure 5A). Both flow cytometry-based cytotoxicity (Figure 5, A and B) and lactate dehydrogenase (LDH) release assays (Figure 5C) demonstrated that TCR-T cells specifically killed tumor cells expressing the HLA-A*11:01 and TP53^R248Q^ mutations and that the cytotoxicity increased dose-dependently as the E:T ratio increased. Furthermore, using the Incucyte real-time dynamic imaging and analysis system, we monitored the killing efficiency of TP53^R248Q^ TCR-T cells over time. The results revealed that TP53^R248Q^ TCR-T cells achieved efficient target cell killing within 48 hours of coculture at E:T ratios of 1:1, 2:1, and 5:1, with cytotoxicity reaching approximately 50%, 60%, and 80%, respectively (Figure 5, D and E). Overall, TP53^R248Q^ TCR-T cells exhibited significantly enhanced cytotoxicity compared with mock CD8^+^ T cells.

We performed additional Incucyte killing assays using human tumor cell lines endogenously expressing the TP53^R248Q^ mutation specifically, NB4 (Figure 5, F and G), CL40 (Figure 5, H and I), and Kasumi-1^A11^ (Supplemental Figure 5, B and C), across a range of E:T ratios. The results consistently demonstrated significantly enhanced killing target tumor cells by TP53^R248Q^-specific TCR-T cells compared with Mock-T cells, further confirming the potent and specific cytotoxic activity of the TCR-T cells against tumor cells expressing the endogenous neoantigen.

To validate clinical relevance, we next evaluated TP53^R248Q^ neopeptide presentation on primary leukemic cells from patients with acute lymphoblastic leukemia at diagnosis (Supplemental Figure 5D). Functional cytotoxicity assays confirmed efficient killing of TP53^R248Q^/HLA-A*11:01–positive primary leukemia cells at E:T ratios of 1:1 (~30%) and 5:1 (~50%), respectively (patient 3; Figure 5J). Conversely, no cytotoxicity of TP53^R248Q^ TCR-T cells was detected against TP53^R248Q^-negative/HLA-A*11:01–positive (patient 1) or TP53^R248Q^-positive/HLA-A*11:01–negative (patient 2) leukemia cells (Figure 5J). Collectively, these results demonstrate that the TP53^R248Q^-reactive TCR confers CD8^+^ T cells with the capacity to specifically recognize and eliminate both established tumor cell lines and primary tumor cells harboring the TP53^R248Q^ mutation in an HLA-A*11:01–restricted manner.

To validate the safety profile of TP53^R248Q^ TCR-T cells, we conducted safety and functional potency assessments for adoptive cell therapies. Primary human cells (CD3^+^ T cell, CD19^+^ B cell, and CD14^+^ monocytes) and human normal tissue-specific cell lines expressing HLA-A*11:01 encompassing hepatocytes, embryonic lung cells, cardiomyocytes, lung epithelial cells, and embryonic kidney cells were cocultured with TP53^R248Q^ TCR-T cells at E:T ratios of 1:1 for 24 hours (Supplemental Figure 5E). Analysis of cytokine secretion profiles (IL-2/IFN-γ/TNF-α) by ELISA revealed no statistically significant elevation of cytokine secretion profiles in TCR-T cells compared with the mock-T control group, suggesting that the cross-reactivity against nonmalignant HLA-A*11:01^+^ cells was almost negligible (Supplemental Figure 5F).

### Adoptively transferred TP53^R248Q^ TCR-T cells exhibit antitumor activity in vivo.

**Figure 6:**
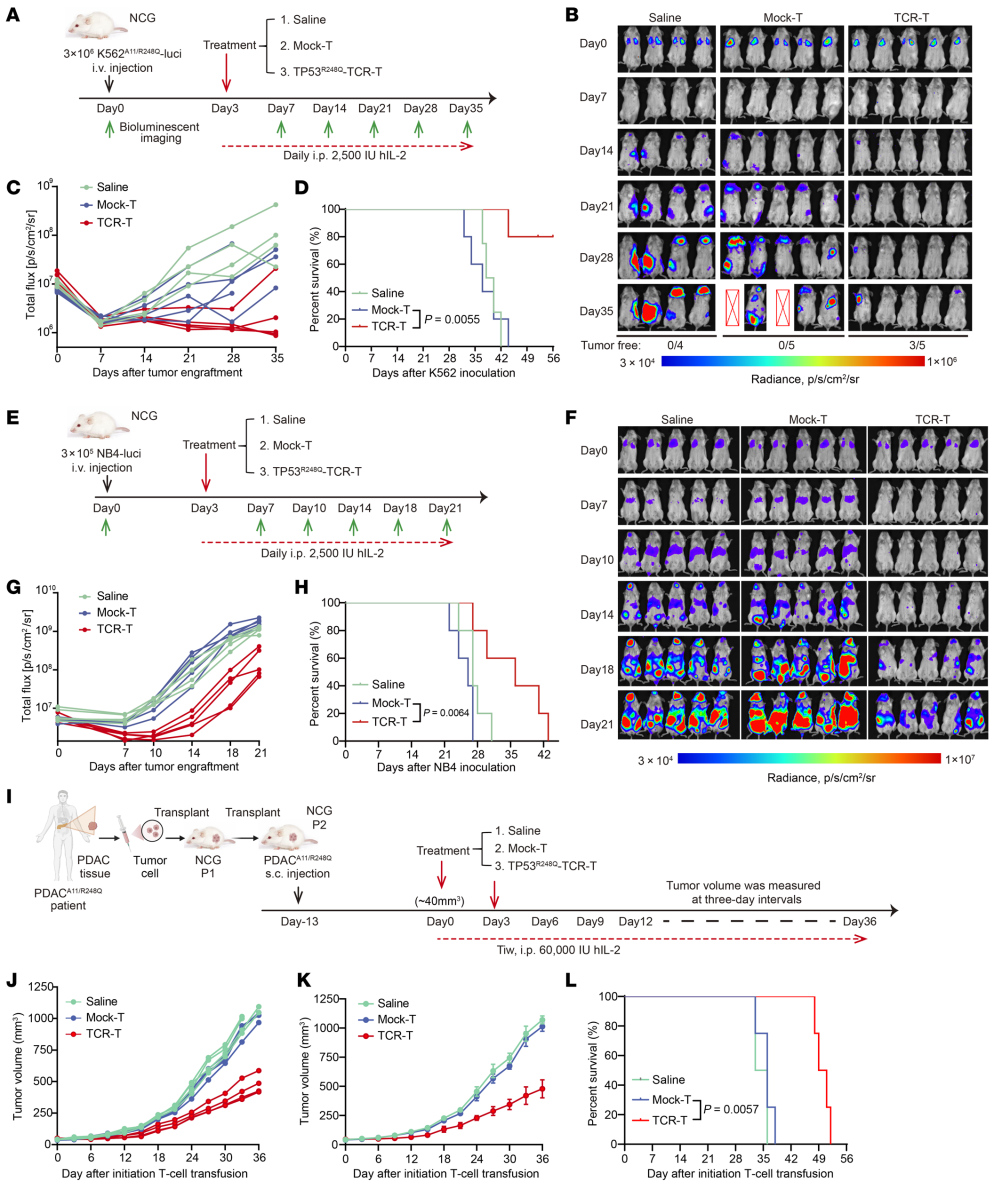
In vivo antitumor efficacy of adoptively transferred T cells introduced with TP53^R248Q^-specific TCR. (**A**) Schematic illustration of the in vivo killing efficacy assessment of TP53^R248Q^ TCR-T cells targeting K562^A11/R248Q^ luciferase tumor cells. (**B**) Bioluminescence imaging was performed on day 0 (+2 h), 7, 14, 21, and 35 after K562^A11/R248Q^ luciferase cell inoculation. (**C**) Tumor burden was quantified as the total flux (photons/s/cm^2^/sr). Each line represents single mouse (saline group, *n* = 4 or different cell treatment groups, *n* = 5). (**D**) Survival curves were analyzed for mice in the saline group (*n* = 4) or different cell treatment groups (*n* = 5). (**E**) Schematic illustration of the in vivo killing efficacy assessment of TP53^R248Q^ TCR-T cells targeting NB4 luciferase tumor cells. (**F**) Bioluminescence imaging was performed on day 0 (+2 h), 7, 10, 14, 18, and 21 after NB4 luciferase cell inoculation. (**G**) Tumor burden was quantified as the total flux (*n* = 5). (**H**) Survival curves were analyzed for mice in the saline group or different cell treatment groups (*n* = 5). (**I**) Schematic representation of the in vivo killing assay of TCR-T cells on the PDX model of PDAC. (**J** and **K**) Individual tumor volume (each line represents single mouse) and mean volume determination of mice in the PDX model (*n* = 4). (**L**) Survival curves were analyzed for mice in the PDX model (*n* = 4). In **D**, **H**, and **L**, log-rank (Mantel-Cox) test was performed to compare survival curves.

Encouraged by the in vitro cytotoxicity results, we next established a cell-derived xenograft model to evaluate the therapeutic efficacy of TP53^R248Q^ TCR-T cells in vivo. Briefly, luciferase-expressing K562^A11/R248Q^ cells were i.v. administered into immunodeficient NCG mice on day 0. Three days posttumor inoculation, mice received i.v. infusion of either 1 × 10^7^ mock-T or TP53^R248Q^ TCR-T cells. Tumor progression was longitudinally monitored by bioluminescence imaging (Figure 6A). Mice in the saline and mock-T cell groups (5/5 mice) exhibited rapid tumor growth and were euthanized at 5 weeks after inoculation owing to severe weight loss and tumor burden. In contrast, 3/5 mice treated with TP53^R248Q^ TCR-T cells remained tumor free, and only 2/5 showed limited tumor progression (Figure 6B). Quantitative biolayer interferometry analysis revealed substantially reduced total and average photon intensities in the TP53^R248Q^ TCR-T group compared with controls (Figure 6C and Supplemental Figure 6, A and B). Upon survival analysis, it was shown that mice in the TP53^R248Q^ TCR-T group had a significantly prolonged the survival period compared with mice in the mock-T group (Figure 6D). These results validate the in vivo efficacy of TP53^R248Q^ TCR-T cells against TP53^R248Q^/HLA-A*11:01–positive tumor cells.

To further confirm the in vivo antitumor activity of TP53^R248Q^ TCR-T cells, we utilized the NB4 cell line, which endogenously express TP53^R248Q^ mutation and HLA-A*11:01 allele, as target cells (Figure 6E). Bioluminescence imaging revealed that TP53^R248Q^ TCR-T cells potently suppressed NB-4 tumor growth compared with mock-T cells or saline (Figure 6F). By week 3, bioluminescence signals in control groups exceeded the detection limit (mean photon intensity, >1.75 × 10^9^), whereas TP53^R248Q^ TCR-T cell–treated mice showed a 9.16-fold reduction (mean intensity, >1.90 × 10^8^) (Figure 6G and Supplemental Figure 6, C and D). Survival analysis further revealed median survival times of 20 days (saline), 21 days (mock-T), and 28 days (TCR-T), underscoring the therapeutic benefit of TP53^R248Q^ TCR-T cell therapy (Figure 6H). Collectively, these results demonstrate that adoptive transfer of TP53^R248Q^ TCR-T cells mediates potent and specific antitumor activity in vivo, supporting its translational potential for HLA-A*11:01–positive cancers harboring TP53^R248Q^ mutations.

To evaluate the therapeutic efficacy of TP53^R248Q^ TCR-T cells in a clinically relevant setting, we established a patient-derived xenograft (PDX) model by s.c. implanting TP53^R248Q^/HLA-A*11:01–positive human pancreatic cancer tissues into immunodeficient NCG mice (Supplemental Table 2, Figure 6I, and Supplemental Figure 6E). When the s.c. tumor volume of the T1 generation mice was about 40 mm^3^, we performed the first round of TCR-T cells as well as mock-T cell infusion and started tumor volume measurements every 3 days as well as intraperitoneal injections of 60,000 U hIL-2 3 times a week. Results demonstrated that TCR-T group mice exhibited delayed tumor progression and reduced mean tumor volume (Figure 6, J and K), with all TCR-T cell–treated mice showing significantly prolonged survival (Figure 6L) compared with the Mock-T or saline group. Flow cytometry results demonstrated that TCR-T cells exhibited progressive dysfunction over time (from week 1 to week 4 after infusion), characterized by a substantially increased expression of exhaustion markers PD-1 and CTLA-4 (Supplemental Figure 6F). Notably, these dynamic changes indicate that T cell functional impairment is closely associated with persistent antigen exposure and the immunosuppressive tumor microenvironment, potentially representing a key mechanism underlying the incomplete tumor clearance observed in PDX model. In summary, these findings conclusively demonstrate that adoptive transfer of TP53^R248Q^ TCR-T cells mediates potent antitumor responses against TP53^R248Q^/HLA-A*11:01–positive tumor in vivo, underscoring its clinical translatability for precision immunotherapy.

## Discussion

Targeting public neoantigens derived from recurrent hotspot driver mutations for treating large patient populations represents a highly promising strategy in TCR-T immunotherapy. In this study, we identified a public neoantigen presented by HLA-A*11:01 allele derived from the TP53^R248Q^ hotspot mutation and successfully isolated a reactive TCR from HD T cell repertoires. The resulting TP53^R248Q^ TCR-T cells demonstrated specific and potent cytotoxicity against tumor cells harboring both the HLA-A*11:01 allele and TP53^R248Q^ mutation in both in vitro and in vivo models. Given the high prevalence of the TP53^R248Q^ mutation across various human malignancies and the approximately 42% frequency of the HLA-A*11:01 allele in the Chinese population (16), we estimate that this therapeutic approach could benefit approximately 40,000–50,000 newly diagnosed patients with cancer annually in China alone (26). An investigator-initiated clinical trial will be conducted to evaluate the safety and efficacy of TP53^R248Q^ TCR-T cell therapy across both hematologic malignancies and solid tumors in our institution.

Despite substantial advances in neoantigen-targeted TCR-T cell therapies, the identification of immunogenic neoantigens and the subsequent screening of reactive TCRs remain major challenges. Here, we developed a strategy combining cmRNA-based polypeptide expression with MAE/MS–based de novo peptide sequencing to systematically characterize neopeptides presented by the designated HLA-I alleles. Compared with traditional neopeptide characterization from tumor tissues or cells, this approach enables high-throughput parallel screening of multiple candidate neopeptides while maintaining enhanced peptide density, facilitating efficient detection in MAE/MS assays. Using this approach, we successfully characterized 2 HLA-A*11:01–restricted neopeptides derived from TP53^R248Q^ (SCMGGMNQR) and TP53^E285Q^ (RTQEENLRK). Among computationally predicted HLA-A*11:01–binding peptides using NetMHC4.1, only 1 TP53^E285Q^-derived neopeptide was experimentally validated via subsequent MAE/MS assay. Conversely, although the TP53^R248Q^ neopeptide was detected in MAE/MS assay, it was not computationally predicted to bind HLA-A*11:01 with sufficient affinity. The relative abundance of hydrophilic amino acids in cancer-related transcripts is a known factor that can reduce the predictive accuracy of algorithms like NetMHCpan for cancer-specific epitopes, which may account for the discrepancy between the weak in silico binding prediction and the strong experimental validation of the SCMGGMNQR peptide (27). Intriguingly, a similar approach reported by Gurung et al. independently identified the TP53^R248Q^ neopeptide (SCMGGMNQR), further validating the robustness and reproducibility of our methodology (28). Currently, we are expanding this approach to identify neoantigens derived from other TP53 hotspot mutations in the context of additional prevalent HLA-I alleles.

Peripheral T cells from HDs have emerged as a valuable source for isolating neoantigen-specific TCRs. In this study, we successfully isolated a reactive TCR targeting the TP53^R248Q^ neoantigen from 1 of 8 screened HDs, suggesting relatively low immunogenicity of this mutation. Nonetheless, the isolated TCR exhibited high specificity and efficacy against TP53^R248Q^-positive tumor cells, in both in vitro and in vivo settings, without detectable cross-reactivity with WT TP53 or other TP53 mutations at the same position. Through an extensive “A” or “G” substitutions assay, we identified an “xxMGGxxQR” motif as critical for TP53^R248Q^ TCR recognition. It is noteworthy that a systematic screen of the human proteome revealed that no endogenous peptide containing this motif activated TP53^R248Q^ TCR-T cells. As a result, no detectable off-target activity of TP53^R248Q^ on TCR-T cells were observed in our study. While these findings suggest a favorable safety profile, thorough evaluation of TP53^R248Q^ TCR-T cell safety remain essential and will be a key focus of future clinical studies.

Several TP53 hotspot mutations, including TP53^R175H^, TP53^Y220C^, and TP53^R248W^, have been reported as immunogenic when presented by HLA-A*02:01 and HLA-A*68:01, respectively (14). In this study, we identified an immunogenic epitope derived from the TP53^R248Q^ mutation restricted by HLA-A*11:01. Our experimental platform demonstrates substantial potential for characterizing the immunogenicity of additional TP53 hotspot mutations in the context of other prevalent HLA-I alleles beyond HLA-A*11:01 and HLA-A*02:01. Interestingly, a recent study by Hoyo et al. revealed an evolutionary balance in TP53 hotspot mutations between protumorigenic functional loss and immunogenic neoantigen presentation (29). In their study, they demonstrated that the TP53^R175H^ mutation, the most functionally WT-like hotspot, exhibits poor HLA-binding capacity, whereas TP53^R248Q^ mutations with near-complete transcriptional inactivation show heightened immunogenicity. Whether these different tumorigenic and immunogenic profiles among TP53 hotspot mutations impact TCR-based immunotherapy efficacy remains unclear. It is important to acknowledge that the use of immunodeficient mouse models, while essential for evaluating human-specific T cell/tumor interactions without confounding alloreactive responses, does not fully recapitulate the complexities of an intact immune system. Future studies should include both preclinical in vivo investigations and clinical trials to systematically evaluate and compare the therapeutic efficacy of TCR-T cell therapies targeting different TP53 hotspot mutations.

While current TP53 mutant-targeting TCRs primarily focus on HLA class I–restricted epitopes, emerging evidence suggests that TP53 mutant-derived neopeptides may also be presented by HLA class II molecules and recognized by CD4^+^ T cells (14). Beyond their conventional helper and regulatory roles, CD4^+^ T cells have demonstrate direct cytotoxic activity against tumors and virus-infected cells (30–32). These findings highlight the therapeutic potential of identifying HLA-II–restricted TP53 mutant neopeptides and isolating their cognate TCRs, particularly for cancers exhibiting both TP53 mutations and HLA-II expression. Notably, recent studies have shown that transgenic expression of HLA-I–restricted TCR coupled with CD8αβ can redirect CD4^+^ T cells to recognize HLA-I–presented neopeptides, conferring direct cytotoxic capacity against HLA-I–expressing tumor cells in both in vitro and in vivo setting (33, 34). Therefore, it is imperative to explore combinatorial strategies using both CD4^+^ and CD8^+^ TCR-T cells with HLA-I–restricted TCR and CD8 transduction to maximize antitumor efficacy in future clinical trials.

In contrast to hematopoietic malignancies, solid tumors establish a profoundly immunosuppressive microenvironment that promotes T cell dysfunction and exhaustion. Analysis of our in vivo PDX model revealed that prolonged exposure to the tumor microenvironment following TP53^R248Q^-TCR-T cell administration led to progressively elevated expression of exhaustion markers, particularly PD-1 and CTLA-4. These findings indicate that T cell functional status, substantially influenced by the duration of antigen exposure, represents a critical determinant of therapeutic outcome. This challenge is further compounded by diverse tumor immune evasion mechanisms, extending beyond the HLA loss variants documented in clinical trials of TP53^R175H^-TCR-T cell therapy (9) to include potential defects in antigen processing and presentation machinery. To address these limitations, numerous strategies — including immune checkpoint blockade, synthetic switch receptors, and therapeutic vaccines — have been investigated to restore antitumor T cell function (35). Rather than undermining the validity of TP53 as a therapeutic target, our findings illuminate the universal challenges in solid tumor immunotherapy and emphasize the necessity of optimized treatment regimens to achieve durable clinical responses.

In summary, our findings establish the immunogenicity of the TP53^R248Q^ hotspot mutation and demonstrate in preclinical models that TP53^R248Q^-specific TCR-T cells constitute a highly specific and potent candidate strategy, thereby supporting the development of future targeted therapies. Moreover, these results provide insights into the validation framework for actionable neoantigen targets in TCR-T cell therapy development.

## Methods

### Sex as a biological variable.

In the clinical portion of this study, HDs and patients of both sexes were included. In the animal experiments, only female mice were used, and it remains unclear whether the findings are applicable to male mice.

### Cell lines, primary cells, and animals.

Jurkat T and hematological tumor cells (K562, NB4, and Kasumi-1) used in this study were cultured in RPMI-1640 medium containing 10% (v/v) FBS; CL40 cells were cultured in IMDM medium containing 20% FBS; HL-7702, MRC-5, AC16, BEAS-2B, OVCAR3, HCC70, and 293T cells were cultured in DMEM medium containing 10% FBS; HL-7702, MRC-5, AC16, BEAS-2B, and CL40 was purchased from Shanghai Jinyuan Biotechnology Company; OVCAR3 and HCC70 were purchased from Shanghai STEMRECELL Biotechnology Company; 293T, Jurkat T, K562, NB4, and Kasumi-1 were maintained in our laboratory. Human-derived primary T cells were cultured in X-VIVO medium (Lonza) containing 10% FBS, and the above medium was supplemented with penicillin-streptomycin solution at a final concentration of 1×; human-derived monocytes and dendritic cells were cultured in ImmunoCult-ACF Dendritic Cell Medium (STEMCELL Technologies). All cells were cultured in 5% concentration of CO_2_ at 37°C in a constant temperature cell culture incubator. NOD/ShiLtJGpt-*Prkdc^em26Cd52^Il2rg^em26Cd22^*/Gpt (NCG) mice (female, 4–6 weeks old) were purchased from GemPharmatech Co. Ltd. All the mice were maintained under specific pathogen–free conditions. Animal studies were approved by the Institutional Animal Care and Use Committee (IACUC, 2021AW041), Shanghai General Hospital.

### PBMC collection and HLA typing.

HDs signed informed consent before donating granulocyte-colony stimulating factor–mobilized peripheral blood mononuclear cells (PBMCs), and PBMCs were isolated by density-gradient centrifugation using lymphocyte separation medium (Corning) and cryopreserved until ready for use. Blood specimen collection involving HDs was approved by the hospital ethics committee and informed consent was signed. High-resolution genomic HLA typing for HDs or patients with PDAC was performed by Tissuebank Biotechnology.

### Circular tandem TP53 mutant mRNA synthesis.

TP53 mutant (R175, Y220, G245, R248, R273, R282, and E285) minigenes were designed and optimized, and the flexible spacer sequences (HHA, HHD, HHHA, HHHHA, HHHHP) were added between minigenes (36). Subsequently, the above minigene mRNA sequences were linked back and forth in tandem to form a single mRNA, and TP53 cmRNA was prepared by cyclization. DNA synthesis and gene cloning were customized and performed by Genewitz. Linear mRNA were synthesized by in vitro transcription from linearized plasmids using the Purescribe T7 High Yield RNA Synthesis Kit (CureMed biotech). For the generation of cmRNA, DNA fragments containing internal ribosome entry sites (IRES), coding regions, and other elements were chemically synthesized and cloned into a linearized pUC57 plasmid digested with a restriction enzyme. cmRNA precursors were synthesized by in vitro transcription from a linearized plasmid using a Purescribe T7 High Yield RNA Synthesis Kit (CureMed biotech). RNA was then purified, after which guanosine triphosphate (GTP) was added to a final concentration of 2 mM along with a buffer including magnesium (Thermo). cmRNA was analyzed using 1% agarose gel electrophoresis and a ssRNA ladder (Thermo) was used as a standard. For HPLC, RNA was loaded onto a 30 mm to 300 mm size exclusion column with a particle size of 5 mm and a pore size of 1,000 Å (Sepax Technologies) on an SCG (Sepure Instruments) protein purification system (Sepure Instruments). Then, the column was eluted with RNase-free phosphate buffer (pH = 6.0), and chromatography was performed at a flow rate of 15 mL/min. RNA with high purity was collected by peak capture and concentrated, and the buffer was replaced with RNase-free water following ultra-centrifugation.

### MAE/MS identifies TP53 antigenic peptide.

For K562 cell lines, K562-HLA stably transfected cell lines with high frequency HLA subtype genes (HLA-A*11:01, HLA-A*24:02, HLA-A*02:01) and then electrotransfected TP53 mutant tandem cmRNA. After 48 hours of electrotransformation, the cell suspension was washed with PBS, and then the HLA protein–bound peptides were eluted from the cell membrane surface using sodium citrate solution (pH = 3.3). The Orbitrap Fusion (Thermo Scientific) was coupled online to an ultra-high-performance LC (1290 Infinity, Agilent) via a nanoelectrospray ion source (Nanospray Flex, Thermo Scientific). Samples were loaded at a flow rate of 5 μL/min for 5 minutes onto a 2 cm × 100 μm C18 trapping column packed in-house with C18 beads of 3 μm diameter (ReproSil-Pur C18-AQ, Dr. Maisch GmbH). Peptides were then separated on a 50 cm × 75 μm column in-house filled with C18 beads of 2.7 μm diameter (Poroshell 120 EC-C18, Agilent). Separation was run at 21°C using a flow rate of approximately 300 nL/min and a 90-minute gradient ranging from 5.6% CH_3_CN to 32% CH_3_CN in 0.1% HCOOH. MS1 scans were acquired at a resolution of 60,000 at 200 Th, and the peptide sequence was analyzed using de novo sequencing to identify the peptides with mutations.

For PDAC-PDX tissue, PDX tumor tissues from patients with PDAC (HLA-A*11:01^+^/TP53^R248Q+^) were mechanically minced and subjected to enzymatic digestion for 1 hour at 37°C with constant shaking in a digestion cocktail containing of 2 mg/mL collagenase IV, 200 μg/mL dispase, and 1 μg/mL DNase. The resulting cell suspension was filtered through a 70 μm strainer to obtain a single-cell suspension, which was subsequently subjected to MAE/MS analysis, and the peptide sequence was analyzed using de novo sequencing to identify the peptides with mutations.

### pMHC-stability assay.

HLA-A*11:01 bound to TP53^R248Q^ (SCMGGMNQR) or TP53^R248WT^ (SCMGGMNRR) and conjugated to Biotin were prepared in our laboratory in-house. The pMHC-stability assay was performed as previously described with minor modifications (37, 38). Streptavidin magnetic beads (Beyotime) were washed twice with wash buffer (DPBS, 0.05% Tween-20, with 0.1% BSA). The peptide–HLA monomer was coupled with prewashed beads for 10 minutes at a ratio of 16.8 × 10^6^ pMHC molecules per microsphere, at a pMHC concentration of 250 nM. Following coupling, the beads were washed and resuspended in 200 μL wash buffer. To monitor complex stability, 20 μL beads were collected at 0, 3, 6, 12, and 24 hours of incubation at 37°C. At each time point, the collected beads were stained with 10 μL of PE-conjugated anti-β2M antibody for 10 minutes. All samples were analyzed immediately after staining using a flow cytometer.

### In vitro stimulation of antigen-specific TCR-T cells.

First, we selected HDs with the appropriate HLA subtypes and isolated monocytes and naive T lymphocytes from the peripheral blood of HDs using CD14^+^ and Naive CD8^+^ T cell sorting kits. CD14^+^ monocytes were used for the differentiation of moDCs, and naive T lymphocytes were used for the screening of TP53 mutant-specific T lymphocytes. The expansion and screening strategy was as follows: the mutant antigen peptide (5 μg/mL) was rotationally incubated with moDCs (2 × 10^6^/mL) for 3 hours at 37°C in a cell culture incubator, and then the moDC-loaded peptide was irradiated (40 Gy) and cocultivated with naive CD8^+^ T lymphocytes from the same donor in a ratio of DC/T = 1:2.5 to activate TP53 antigen-specific T lymphocytes, and 30 ng/mL IL-21 (Peprotech) was added. On day 2, the culture was continued by replacing 1640 medium containing IL-2 (25 U/mL), IL-7 (10 ng/mL), and IL-15 (10 ng/mL) (Peprotech); subsequent half-exchanges were performed every 3 days; and the culture was supplemented with fresh IL-2, IL-7, and IL-15 cytokines. On day 14, fresh, irradiated-treated moDCs loaded with mutant antigen peptides were reprepared for a second round of stimulation of TP53 antigen-specific T cells. At day 24, a third round of stimulation of TP53 antigen-specific T cells was performed. At day 25 of coculture, TP53 mutation–specific T cells were detected after staining using CD8 antibody and HLA-A*11:01–TP53^R248Q^ tetramer.

### Flow cytometry.

For extracellular staining, cells were collected by centrifugation and subsequently resuspended into 100 μL of suspension FACS buffer, followed by the addition of antibodies for extracellular staining and for cell surface antibody labeling, gentle mixing, and incubation on ice or at 4°C for 30 minutes for full staining. After staining, FACS buffer was added, cells were centrifugated at 300*g* for 5 minutes to discard the supernatant, and the washing step was repeated 2–3 times. Subsequently, the cells were resuspended by adding FACS buffer and subsequently detected on a BD LSR Fortessa flow analyzer or sorted and collected from specific subpopulations using a BD Aria III sorting flow cytometer.

For intracellular staining, after extracellular staining, cells were washed with staining buffer and then fixed and permeabilized with the BD Cell Fixation/Permeabilization solution kit according to the manufacturer’s instructions (BD Biosciences). Cells were washed with perm/wash solution, followed by intracellular staining with intracellular antibody (30 minutes, room temperature). Finally, cells were washed and resuspended with perm/wash buffer. Samples were detected on a LSR Fortessa (BD Biosciences). Analysis was performed using FlowJo software (version 10.8).

The flow cytometry antibodies used in this study are listed in the Supplemental Methods.

### Tetramer labeling assay.

HLA-A*11:01–tetramers bound to TP53^R248Q^ (SCMGGMNQR) and conjugated to PE were prepared in our laboratory in-house. Cells were labeled with PE-conjugated tetramer for 20 minutes at room temperature, followed by extracellular antibodies against CD8 and live/dead dye for an additional 20 minutes at 4°C. After staining, cells were washed 3 times with FACS buffer (DPBS supplemented with 2% FBS) and detected on a BD LSR Fortessa flow cytometer or cells were sorted on BD ARIES III flow cytometer.

### Single-cell TCR and transcription sequencing.

FACS-sorted CD8^+^tetramer^+^ cells were harvested, final cell concentration was determined by cell count on a hemocytometer, and the cell concentration was adjusted to approximately 1,000 cells per microliter with a cell viability greater than 98%. Sorted CD8^+^/tetramer^+^ T cells were prepared for 10× Genomics single-cell sequencing of rearranged VDJ according to manufacturer’s instructions. Detailed quality control metrics were generated and evaluated using single-cell analysis packages from Bioconductor, including DropletUtils, scater, and scran. Genes detected in fewer than 3 cells, and cells where fewer than 200 genes had non-0 counts were filtered out and excluded from subsequent analysis. Low-quality cells with more than 15% of the read counts derived from the mitochondrial genome were also discarded. After single-cell sequencing and analysis, the TCR sequences were evaluated using the Loupe VDJ browser (Cell Ranger, 10× Genomics). The top 10 rankings were performed according to the frequency of TCRs, and the top 3 ranked human TCR sequences were selected for subsequent functional experiments.

### Plasmids and lentiviral transduction.

pCMV-dR8.91, pVSVG, and pCDH-luciferase-puro plasmids were purchased from Unibio Biotechnology. pEF1a-TP53^R248Q^-IRES-mCherry, pEF1a-TP53^WT^-IRES-mCherry, pHR-SFFV-HLA-IRES-GFP (A*11:01, A*02:01, A*24:02), pHR-SFFV-TP53^R248Q^-TCR (clones 1, 2, and 3) plasmids were synthesized by Genscript Biotechnology. HEK293T cells were transfected with pHR-SFFV or pEF1a transfection plasmids, pCMV-dR8.91 (packaging plasmid) and pVSVG (envelope plasmid) using Opti-MEM reagent (Gibco). Forty-eight hours after transfection, lentiviral supernatants were collected, filtered through 0.45 microporous membrane filters, concentrated by ultracentrifugation, and frozen in aliquots at –80°C prior to use.

### Human T cell culture and lentiviral transduction.

Human CD3^+^ T cells were isolated from PBMCs of HDs with EasySeq Human CD3 Positive Selection Kit II (STEMCELL Technologies). CD3^+^ T cells were cultured in RPMI-1640 medium supplemented with 10% FBS and 500 U/mL hIL-2 protein (PeproTech). CD3^+^ T cells were stimulated with Dynabeads Human T-Expander CD3/CD28 (Thermo) at a bead-to-cell ratio of 2.5:1 for 48 hours before viral infection. 3 × 10^6^ T cells were resuspended in 2 mL viral supernatant containing 500 U/mL IL-2 and 5 μg/mL protamine sulfate. Cells were plated into wells of 6-well plates. Cells were centrifuged for 90 minutes at 800*g*, 30°C, for spinfection and incubated overnight at 37°C, 5% CO_2_. Medium was carefully aspirated from each well, and 1 mL of viral supernatant containing 5 μg/mL protamine sulfate was added. Cells were centrifuged for 90 minutes at 800*g*, 30°C, for spinfection and incubated overnight in the cell culture chamber. The supernatant was discarded after centrifugation and resuspended with 1640 medium containing IL-2 (500 U/mL) to final volume of 4 mL/well. Infection efficiency was detected by flow cytometry on days 2 after 2 rounds of lentiviral infection.

### TP53^R248Q^ TCR-T cell in vitro activation assays.

For the Jurkat T-NFAT-luciferase reporter system, viable cells were counted and the cells were harvested. Cells were plated at a density of 5 × 10^4^ cells/well in 96-well culture plates (Round Bottom) in 40 μL 1640 medium. According to E:T = 1:1, TP53^R248Q^ TCR-T cells were inoculated with K562^A11^ or K562^A11/R248Q^ cells. Cells were incubated at 37°C with 5% CO_2_ for 12 hours. 80 μL of ONE-Glo Luciferase reagent (Promega) was added per well at room temperature for 2 minutes and then 110 μL was transfered to a corresponding 96-well Bioluminescence detection plate, follow by detecting the RLU of the plate using a luminescence plate reader.

For the detection of T cell activation–related markers and cytokines, K562^A11^ or K562^A11/R248Q^ cells were prepared by lentiviral infection of K562 cells; TCR-T cells were inoculated into round-bottomed 96-well plates with TCR-T cells and K562 control or target cells according to E:T ratio = 1:1. After 24 hours of coculture, extracellular staining was performed, and the expression levels of CD8^+^ T cell activation-related markers, such as CD25, CD69 CD107a, and CD137, as well as the expression levels of cytokines IL-2, TNF-α, GzmB and IFN-γ were detected by flow cytometry, in order to assess whether the K562^A11/R248Q^ target cells could specifically activate in vitro TP53^R248Q^ TCR-T cells.

For enzyme linked immunospot assay (ELISpot), moDCs or K562-HLA cell lines were loaded with TP53^R248WT^ (SCMGGMNRR) or TP53^R248Q^ (SCMGGMNQR) peptides (Purities > 95%), respectively, and cocultured with primary CD8^+^ T or TP53^R248Q^ TCR-T effector cells in ELISpot PVDF plate. After 24 hours of coculture, the cells were washed 5 times with 200 μL DPBS to completely remove the cells, 100 μL of 1 μg/mL detection antibody 7-B6-1-biotin (Mabtech) was added to the culture system, and the cells were incubated at 37°C for 2 hours. The cells were then incubated with 100 μL streptavidin. Subsequently, 100 μL streptavidin-HRP was added and incubated at room temperature for 1 hour. After 5 washes with 200 μL of DPBS, 100 μL of TMB was added to each well until complete spots appeared in the wells, followed by the addition of sterile water to interrupt the color development process. After the plates were allowed to dry naturally overnight, the IFN-γ ELISpot results were analyzed and quantified using the CTL ELISpot reader.

For ELISA, human IL-2 (#3445-1HP-1), human IFN-γ (#3420-1HP-1), and human TNF-α (#3512-1HP-DP-1) levels were quantified using commercial ELISA kits (all Mabtech) following the manufacturer’s protocols. 1 × 10^5^ K562^A11^, K562^A11/R248Q^ cells, primary human cells (CD3^+^ T cell, CD19^+^ B cell, and CD14^+^ monocytes), or human normal tissue-specific cell lines expressing HLA-A*11:01 were cocultured with mock-T or TP53^R248Q^ TCR-T effector cells in 96-well culture plates (round bottom) according to the E:T = 1:1. 96-well plates precoated with capture antibodies were blocked with 1% BSA/PBS for 1 hour at 25°C. After 24 hours of coculture, 100 μL cell culture supernatant was added in triplicate and incubated for 2 hours at 37°C. After 5 washes with 0.05% Tween-20/PBS, biotinylated detection antibodies (1:200 dilution) were added for 1 hour, followed by streptavidin-HRP (1:2,500) for 30 minutes. TMB substrate (Thermo Fisher) was added for 15 minutes, and reactions were stopped with 2 N H_2_SO_4_. Absorbance at 450 nm (reference 570 nm) was measured using a Biotek microplate reader. Standard curves (7.8–500 pg/mL) were generated via 4-parameter logistic regression (R^2^ > 0.99).

For TCR-T EC_50_ detection, K562^A11^ cells were loaded with different concentrations of TP53^R248Q^ neopeptide or TP53^R248WT^ peptide at different concentrations of 10^–5^ M to 10^–14^ M, respectively. TP53^R248Q^ TCR-T cells were inoculated into round-bottom 96-well plates with K562^A11^ cells at E:T = 1:1, respectively. Following 24 hours of coculture, cells in the coculture system were collected and changes in the expression levels of CD69 and CD137 of TP53^R248Q^ TCR-T cells were detected by flow cytometry in order to assess the EC_50_ values associated with the activation of TP53^R248Q^ TCR-T cells.

### Detection of TP53^R248Q^ TCR-T cell specificity.

For the alanine/glycine substitution and glutamine mutation scanning assays, K562^A11^ cells were inoculated with TP53^R248Q^ antigenic peptide (SCMMGGNQR) by loading K562^A11^ with alanine (A), glycine (G) or glutamine (Q) at different positions (aa positions 1 to 9) and then inoculated with TP53^R248Q^ TCR-T cells with “A,” “G,” or “Q” substituted TP53^R248Q^ antigenic peptide (SCMMGGNQR), respectively, according to the E:T ratio = 1:1. TP53^R248Q^ TCR-T cells were cocultured with K562^A11^ cells loaded with “A,” “G,” or “Q” substituted TP53^R248Q^ antigenic peptide at E:T = 1:1. After 24 hours of coculture, cells were collected from the coculture system and subsequently flow cytometry was detected for CD69 and CD137 expression levels of TP53^R248Q^ TCR-T cells.

For the activation detection of similar motifs, after analyzing the human database through the PROSITE database (https://prosite.expasy.org/), a total of 9 peptide sequences of 4 proteins (CD247, ssDNA binding protein, stromalin-3, and stromal antigen 3) were found to be consistent with the “xxMGGxxQR” motif characteristics. After loading K562^A11^ cells with the different similar peptides mentioned above, TP53^R248Q^ TCR-T cells were cocultured with K562^A11^ cells by E:T = 1:1 inoculation, and then the expression levels of CD69 and CD137 in TP53^R248Q^ TCR-T cells were detected by flow assay.

### SPR analysis of TP53^R248Q^-tcr binding kinetics and affinity.

TCR β chain-HA tag and TCR α chain-His tag genes were synthesized and cloned into pTT5 plasmid. Plasmids were transiently cotransfected into HEK293 cells. Following a 3-day culture period, the culture medium was harvested and purified by Nickel column, and final product was dissolved in PBS buffer. 1×HBS-EP buffer (10 mM HEPES, 150 mM NaCl, 3mM EDTA, 0.05% surfactant P20) was used as the running buffer for experimental operation. At the flow rate of 10 μL/min, Human Peptide Ready HLA-A*11:01 Monomer, His-Avi Tag with SCMGGMNQR peptide complex (Kactus Biosystems) was diluted 10 μg/mL to captured on flow cell 3 of CM5-SA chip, and the capture level was about 3,615 RU. Flow cell 1 was used as a blank reference channel. TP53^R248Q^-TCR at 1 concentration per injection cycle was measured for association and dissociation with Human Peptide Ready HLA-A*11:01 Monomer, His-Avi Tag with SCMGGMNQR peptide complex. At the flow rate of 30 μL/min, the diluted TP53^R248Q^-TCR was sequentially injected into flow cells at the concentration of 250–8,000 nM (double dilution), the association time was 60 seconds, and the dissociation time was 90 seconds. Using Biacore T200 Evaluation Software (version 3.2), TP53^R248Q^-TCR and HLA-A*11:01 Monomer, His-Avi Tag with SCMGGMNQR peptide complex was analyzed by 1:1 binding mode, where K_on_ is the association rate constant, K_off_ is the dissociation rate constant, and K_D_ is the affinity constant.

### TP53^R248Q^ TCR-pMHC complex structure prediction.

Complex structure of TP53^R248Q^ TCR binding TP53^R248Q^/HLA-A*11:01 (pMHC) or TP53^R248Q^ TCR binding TP53^R248WT^-HLA-A*11:01 were predicted by Alphafold3 (https://alphafoldserver.com/). Hydrogen bonds between TP53^R248Q^ neopeptide and surrounding residues (HLA-A*11:01 and TP53^R248Q^ TCR) were generated using ChimeraX. Using Alphafold3, we generated a molecular model with high interface confident score.

### TP53^R248Q^ TCR-T cell in vitro killing assays.

For flow cytometry detection, the K562^A11^, K562^A11/R248Q^, NB4 cell lines or primary acute lymphoblastic leukemia cells were gently resuspend in the CellTrace violet dye solution (Thermo), incubated for 20 minutes at room temperature, and protected from light. The cells were pelleted by centrifugation and resuspended in fresh, prewarmed complete culture medium. Then, mock-T or TP53^R248Q^ TCR-T effector cells and target cells were inoculated into round-bottom 96-well plate at an E:T cells ratio of 1:1, 2:1 or 5:1, respectively. After 24 hours of coculture, the CellTrace violet^+^/eFluor780^+^ (LIVE/DEAD) ratio was detected by flow cytometry to evaluate the target cell lysis (%).

For LDH assay, after target cells were killed by T lymphocytes, the cell membrane ruptured and LDH in the cytoplasm was released into the peritoneal fluid. By detecting the activity of LDH, the killing ability of TCR-T cells can be detected. TCR-T cells were cocultured with the CL40 cell line expressing the corresponding HLA gene and TP53 mutant gene according to different targeting ratios and cocultured with TCR-T cells at 37°C for 24 hours. The supernatant in the coculture system was collected, and the enzyme activity and release of LDH in the culture medium were detected by colorimetric assay to calculate the killing efficiency of TCR-T cells on target cells. Data were normalized to maximal Triton X-100 lysis.

For Incucyte live-cell imaging, TP53^R248Q^ TCR-T cells or mock CD8^+^ T cells with K562^A11^, K562^A11/R248Q^, NB4, CL40, Kasumi-1, or Kasumi-1^A11^ tumor cells were added to 96-well plates for coculturing according to the E:T ratios of 1:1, 2:1, and 5:1, respectively. The Incucyte Annexin V Dyes reagent (Sartorius) could then be diluted in complete medium to a final dilution of 1:200. The 96-well plate containing the cells was placed into the Incucyte Live Cell Assay Instrument (Sartorius), and apoptosis was monitored using the appropriate fluorescent channel. Specific parameters were as follows: observation magnification, ×20 objective; channel selection, green|red|orange; and scanning interval, every 1 hour until the killing efficiency assay was completed after 48 hours of coculture. At the end of the assay, the in vitro killing efficiency of TP53^R248Q^ TCR-T cells was analyzed using Incucyte Live Cell Analysis System software.

### TP53^R248Q^ TCR-T cell in vivo killing assay.

For cell-derived xenograft model: 4~6-week-old NCG female mice (GemPharmatech) were selected, and 3 × 10^6^ K562^A11/R248Q^ luciferase cells or 3 × 10^5^ NB4 luciferase cells were injected into the mice via the lateral tail vein. Three days after transplantation of the tumor cells, 1 × 10^7^ TCR-T cells were injected via the lateral tail vein, and mock-T lymphocytes, which had not been transfected with the TCR gene, were used as a negative control. The total photon number intensity as well as the average photon number intensity of the mice were analyzed by in vivo bioluminescence imaging once a week or twice a week after tumor cell inoculation to assess the in vivo killing efficacy of TP53^R248Q^ TCR-T cells on target tumor cells.

For PDX model, To evaluate the therapeutic efficacy of TP53^R248Q^ TCR-T cells in a clinically relevant setting, we established a PDX model by s.c. implanting TP53^R248Q^/HLA-A*11:01–positive human pancreatic cancer tissues into immunodeficient NCG mice. Briefly, fresh PDAC tissues were obtained from patients with confirmed TP53^R248Q^/HLA-A*11:01–positive tumors under Institutional Review Board–approved protocols and with written informed consent. Tumor tissues were minced into 10 mm³ fragments in ice-cold RPMI-1640 supplemented with 1% penicillin/streptomycin and 10% FBS. Fragments were s.c. implanted into 4- to 6-week-old NCG mice using a 12-gauge trocar under isoflurane anesthesia. Engraftment success was monitored weekly by caliper measurements (tumor volume = length × width^2^ × 0.5). First-generation (P1) tumors reaching 800 mm^3^ were harvested, minced, and serially passaged into secondary recipients (P2) to establish stable PDX lines. When the s.c. tumor volume of the P2 generation mice was about 40 mm^3^, we performed the first round of TCR-T cell as well as mock-T cell (1 × 10^7^ per mouse) infusion, respectively, and started tumor volume measurements every 3 days (tumor volume = length × width^2^ × 0.5) as well as intraperitoneal injections of 60,000 U of hIL-2 3 times a week. After the 3 days of initial T cell infusion, we performed the second round of TCR-T cell as well as mock-T cell (1 × 10^7^ per mouse) infusion. Body weight changes, GVHD symptoms, and survival of the mice were recorded daily.

### Statistics.

Results are expressed as mean ± SEM of at least 3 independent experiments performed in triplicate. Sample sizes for in vitro experiments were *n* ≥ 3. Sample sizes for in vivo experiments were *n* ≥ 4. Gaussian distribution was used to test if the samples were normally distributed. If the samples conformed to normal distribution, experiments comparing 2 groups were then analyzed by 1-tailed Student’s *t* test. For experiments comparing 3 or more groups, 1-way ANOVA or 2-way ANOVA was performed, and groups were post hoc compared using Tukey’s multiple comparison test. If the samples did not conform to a normal distribution, a nonparametric test was used and was compared using the Wilcoxon test. For in vivo experiments, survival curve analyses were performed using the log-rank Mantle-Cox test. All statistical analyses were performed using GraphPad Prism version 8.0 software. *P* < 0.05 was considered a statistically significant difference.

### Study approval.

Animal studies were approved by the Shanghai General Hospital’s IACUC (2021AW041). Blood specimen collection involving HDs and patients was approved by the Shanghai General Hospital ethics committee, and written informed consent was signed (K-2023-007).

### Data availability.

The source data for data points shown in graphs and values behind means are provided in the Supporting Data Values file. The original single-cell sequencing files were deposited in the NCBI’s Sequence Read Archive (SRA) database under BioProject accession ID PRJNA1395280. The datasets generated during and/or analyzed during the current study are available from the corresponding authors on reasonable request.

## Author contributions

Conceptualization and experimental design: LS, XS, and Y Zhang. Experimentation and acquisition of data: LS, JX, ZC, QH, C Zhang, XZ, Xiaodan Ding, JF, FL, JZ, and PW. Analysis and interpretation of data: QL, and JL. Technical and material support: MJ, YY, Xueying Ding, Y Zheng, C Zuo, TM, MZ, BM, YW, QG, HZ, and JH. Clinically relevant guidance: BD and LW. Writing of the original paper: LS, PW, and Y Zhang. Supervision: XS and Y Zhang. LS, ZC, and JX are co–first authors of this work. The order of co–first authors (LS, ZC, JX) was determined based on their relative contributions to the study, with the sequence reflecting the extent of each author’s input and effort.

## Funding support

- Prevention and Control of Emerging and Major Infectious Diseases-National Science and Technology Major Project (2025ZD01908300, 2025ZD01908302).
- Science and Technology Commission of Shanghai Municipality of China (23J11900300, 24YF2734200, 19JC1414400).
- National Key R&D Program of China (2021YFC2502300, 2019YFA0111000).
- National Natural Science Foundation of China (82273425, 82473196, 8250010605).
- Shanghai Shen Kang Hospital Development Center, 3-year development project (SHDC2020CR1012B).

## Supplementary Material

Supplemental data

Supporting data values

## Version 1 01/13/2026

In-Press Preview

## Version 2 03/02/2026

Electronic publication
