# Supplementary material for: T cell receptor–engineered T cells targeting the TP53R248Q neoantigen elicit antitumor effects in human cancer models
Source: J Clin Invest. 2026 Jan 13;136(5):e196613. doi: 10.1172/JCI196613 (PMC12948421; doi:10.1172/JCI196613)
Supplement: Supplemental data [file jci-136-196613-s138.pdf]

Supplementary Information

Supplemental Method

Flow Cytometry antibodies

The list of flow cytometry antibodies used in this study for intracellular and extracellular staining is as follows. The following antibodies were purchased from BioLegend Company: BV650-conjugated anti-hCD3 (UCHT1, Cat# 300467), BV510-conjugated anti-hCD8a (RPA-T8, Cat# 301048), PE-conjugated Streptavidin (Cat# 405203), APC-conjugated anti-mTCRβ-C (H57-597, Cat# 109211), FITC-conjugated anti-mTCRβ-C (H57-597, Cat# 109206), BV421-conjugated hCD25 (M-A251, Cat# 356114), PE-conjugated hCD69 (FN50, Cat# 310906), BV421-conjugated hCD69 (FN50, Cat# 310930), APC-conjugated hCD137 (4B4-1, Cat# 309810), FITC-conjugated hCD107a (LAMP-1, Cat# 328606), APC-conjugated hIL-2 (MQ1-17H12, Cat# 503810), APC-conjugated hIFN-γ (4S.B3, Cat# 986702), PE-Cy7-conjugated hTNF-α (MAb11, Cat# 502930), FITC-conjugated Granzyme B (QA16A02, Cat# 372206), BV605-conjugated PD-1 (29F.1A12, Cat# 135220), BV421-conjugated CTLA-4 (BNI3, Cat# 369606), and PE-conjugated β2m (2M2, Cat# 316306). The following antibodies were purchased from ThermoFisher, Inc.: CellTrace Violet (Cat# C34557), LIVE/DEAD™ Fixable Near IR (780) Viability Dye (Cat# L34994).

Supplemental Table 1. Composition of TP53 tandem mutation vectors and its motif sequences

TP53 Site	Mutation frequency	Motif	Sequences	Length
R175	4.80%	WT-R175	YKQSQHMTEVVRRCPHHERCSDSDG	25 aa
		R175H	YKQSQHMTEVVRHCPHHERCSDSDG	
		R175C	YKQSQHMTEVVRCCPHHERCSDSDG	
Y220	1.70%	WT-Y220	DRNTFRHSVVVPYEPPEVGSDCTTI	25 aa
		Y220C	DRNTFRHSVVVPCEPPEVGSDCTTI	
G245	3.12%	WT-G245	HYNYMCNSSCMGGMNRRPILTIITL	25 aa
		G245S	HYNYMCNSSCMGSMNRRPILTIITL	
		G245D	HYNYMCNSSCMGDMNRRPILTIITL	
		G245C	HYNYMCNSSCMGCMNRRPILTIITL	
		G245V	HYNYMCNSSCMGVMNRRPILTIITL	
R248	6.79%	WT-R248	YMCNSSCMGGMNRRPILTIITLEDS	25 aa
		R248Q	YMCNSSCMGGMNQRPILTIITLEDS	
		R248W	YMCNSSCMGGMNWRPILTIITLEDS	
R273	6.55%	WT-R273	SGNLLGRNSFEVRVCACPGRDRRTE	25 aa
		R273H	SGNLLGRNSFEVHVCACPGRDRRTE	
		R273C	SGNLLGRNSFEVCVCACPGRDRRTE	
R282	2.59%	WT-R282	FEVRVCACPGRDRRTEEENLRKKGE	25 aa
		R282W	FEVRVCACPGRDWRTEEENLRKKGE	
E285	0.96%	WT-E285	RVCACPGRDRRTEEENLRKKGEPHH	25 aa
		E285K	RVCACPGRDRRTKEENLRKKGEPHH	
		E285Q	RVCACPGRDRRTQEENLRKKGEPHH	

Supplemental Table 2. Diagnosis and HLA-related information of PDAC patient

Patient ID	Age	Sex	Diagnose	TNM classification	$TP53^{R248Q}$ mutation	HLA-A*11:01
00712708	52	Female	PDAC	T3aN1M0 IIIA	+	+

Supplemental Table 3. HLA typing information for healthy donors

Donor ID	HLA-A	HLA-B	HLA-C	$CD8^{+}$/ A11-$TP53^{R248Q}$-$Tetramer^{+}$
1	11:01,02:01	15:11,54:01	01:02,03:03	<0.001%
2	11:01,11:02	15:27,51:01	04:01,16:02	<0.001%
3	11:01,33:03	56:03,44:03	01:02,07:06	<0.001%
4	11:01,24:02	40:01,54:01	01:02,03:04	<0.001%
5	11:01,03:01	44:02,54:01	01:02,05:01	<0.001%
6	11:01,24:02	13:01,40:06	03:04,14:02	<0.001%
7	11:01,24:02	15:27,51:01	04:01,14:02	<0.001%
8	11:01,02:01	15:18,40:01	03:02,07:02	**0.21%**

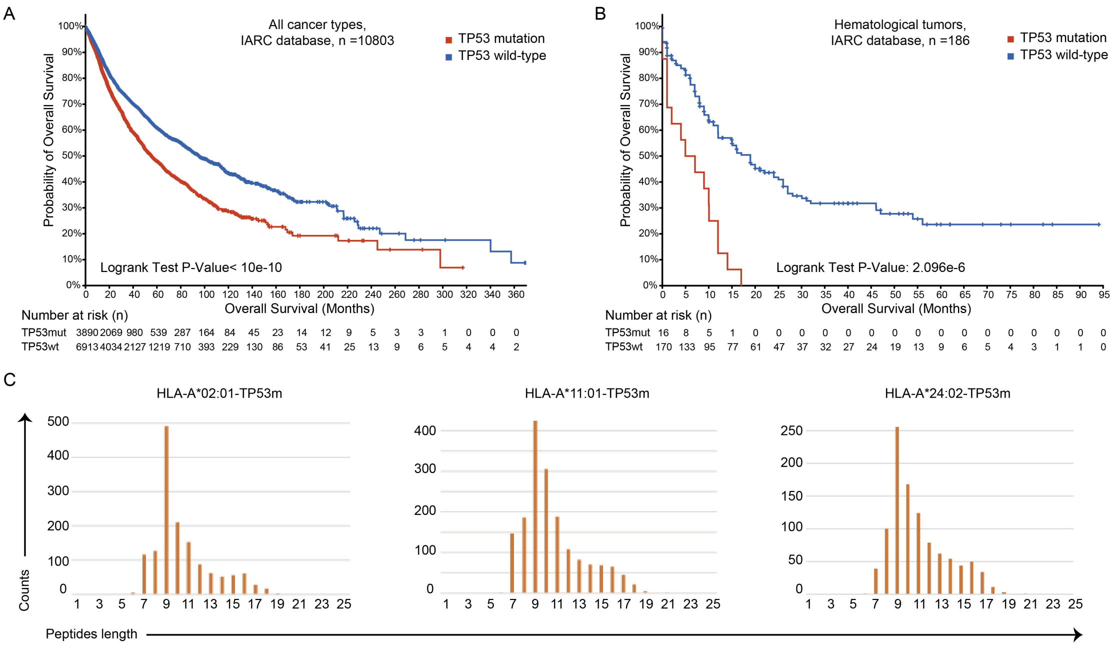

D

Cell type	HLA allele	Transfection	Total peptides	Predicted MHC-I binders	MAE/MS Identified mutant peptide
K562	A*02:01	Tandem-TP53m cmRNA	1468	596 (41%)	R175H:HMTEVVRHC
	A*11:01		1739	622 (36%)	R248Q:SCMGGMNQR; E285Q:RTQEENLRK
	A*24:02		1026	330 (32%)	-

E

Peptide length	HLA-A	TP53 mutant allele	NetMHC predicted epitope	Affinity (nM)	E.L. % Rank	Bind Level	MAE-IP-MS detected
9 mers	A*11:01	G245S	SSCMGSMNR	31.83	0.25	SB	-
	A*11:01	G245D	SSCMGDMNR	168.23	1.00	WB	-
	A*11:01	G245C	SSCMGCMNR	97.22	0.70	WB	-
	A*11:01	G245V	SSCMGVMNR	35.04	0.25	SB	-
	A*11:01	E285K	RTKEENLRK	227.25	1.20	WB	-
	A*11:01	E285Q	RTQEENLRK	71.24	0.25	SB	+
	A*11:01	R248Q	SCMGGMNQR	10938.61	9.00	-	+
10 mers	A*11:01	E285Q	RRTQEENLRK	194.53	1.10	WB	-
	A*11:01	E285Q	RTQEENLRKK	156.93	0.90	WB	-
	A*11:01	R248Q	SSCMGGMNQR	132.92	0.80	WB	-

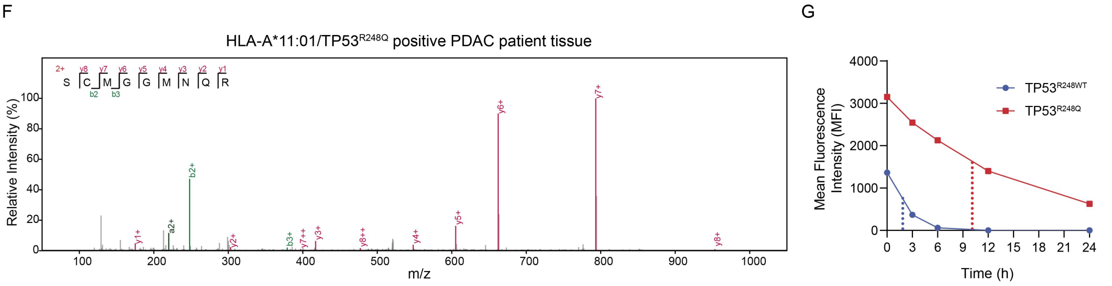

Supplemental Figure 1. MAE-IP-MS data analysis and NetMHC4.1 prediction of HLA-A*11:01 delivery of TP53 mutant peptides. Clinical relevant between TP53 mutation status and patient survival in all cancers (*n*=10803) **(A)** or hematologic

Malignancies (*n*=186) **(B)** from IARC TP53 Database. **(C)** Peptide distribution of monoallelic K562 cells after MAE detected by mass spectrometry. **(D)** Peptide sequences detected by mass spectrometry after MAE in monoallelic K562 cells and their predicted proportions likely to be delivered by MHC-I. **(E)** Summary table listing the NetMHC4.1 predicted TP53 mutant peptides with HLA-A*11:01 binding affinities (eluted ligand % rank/bind level) and corresponding MAE-IP-MS detection status. **(F)** $TP53^{R248Q}$ neo-peptide (SCMGGMN**Q**R) was found to be specifically presented in PDAC patient tissue through MAE-MS. **(G)** Off-rates for $TP53^{R248WT}$ or $TP53^{R248Q}$ peptide binding to HLA-A*11:01 measured by flow cytometry. Vertical lines mark the calculated half-lives for each group; dots show the mean fluorescence intensity (MFI) of intact pMHC complexes on fluorescent particles at the indicated time points.

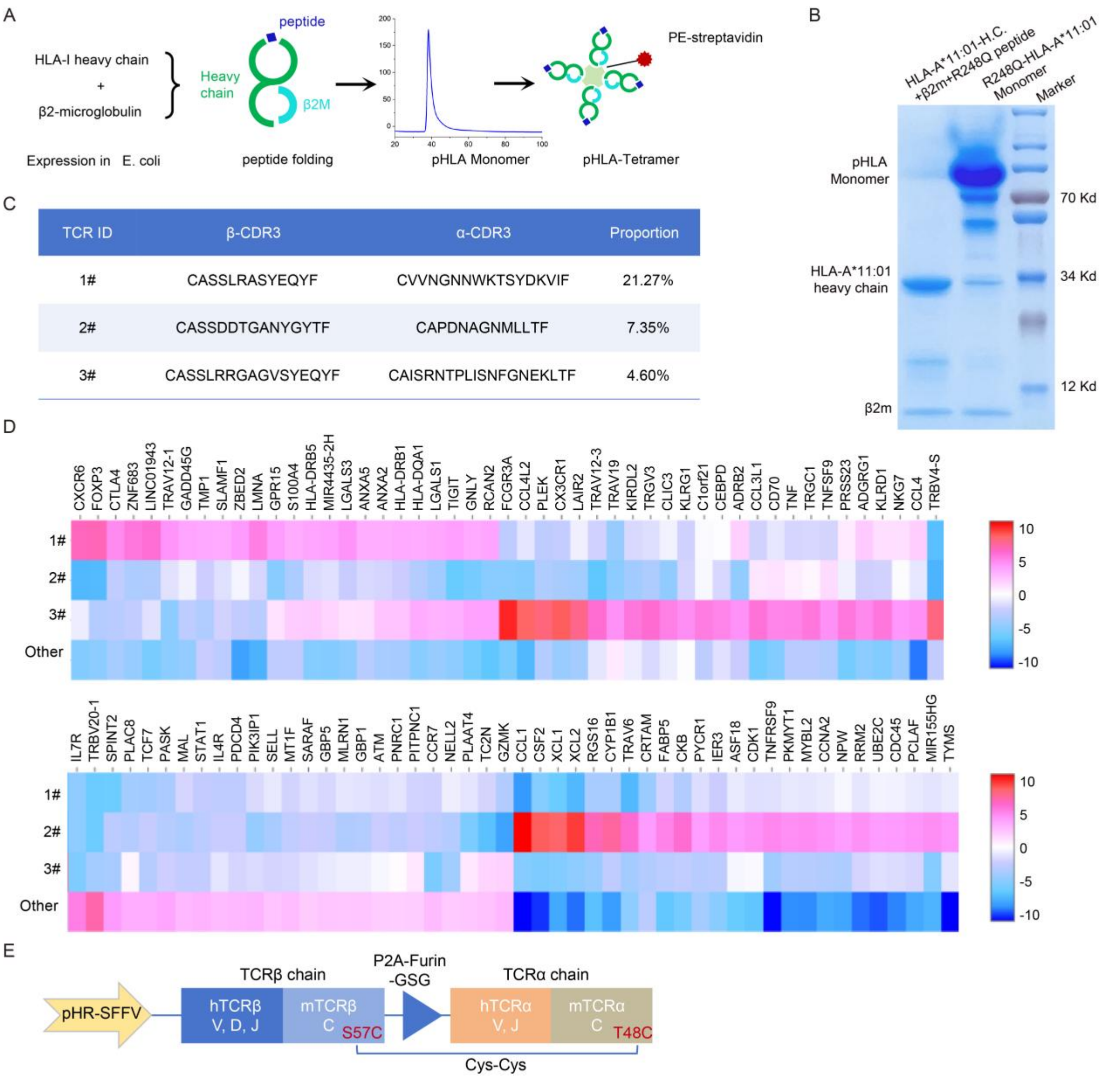

TCR ID	β-CDR3	α-CDR3	Proportion
1#	CASSLRASYEQYF	CVVNGNNWKTSYDKVIF	21.27%
2#	CASSDDTGANYGYTF	CAPDNAGNMLLTF	7.35%
3#	CASSLRRGAGVSYEQYF	CAISRNTPLISNFGNEKLTF	4.60%

Supplemental Figure 2. Preparation of Tetramer, and analysis of data after Tetramer staining combined with single-cell transcriptome sequencing. (A-B) Procedure for the preparation of TP53^{R248Q}/HLA-A*11:01-Tetramer. **(C)** CDR3s sequences and percentages of the top three TCRs after single-cell TCR sequencing. **(D)** Single-cell TCR combined with transcriptome sequencing to analyze the transcriptomic differences between the top three ranked TCR-Ts and other T cells. **(E)** Schematic diagram of TP53^{R248Q} TCR Lenti-vector design, Mutation sites are marked in red.

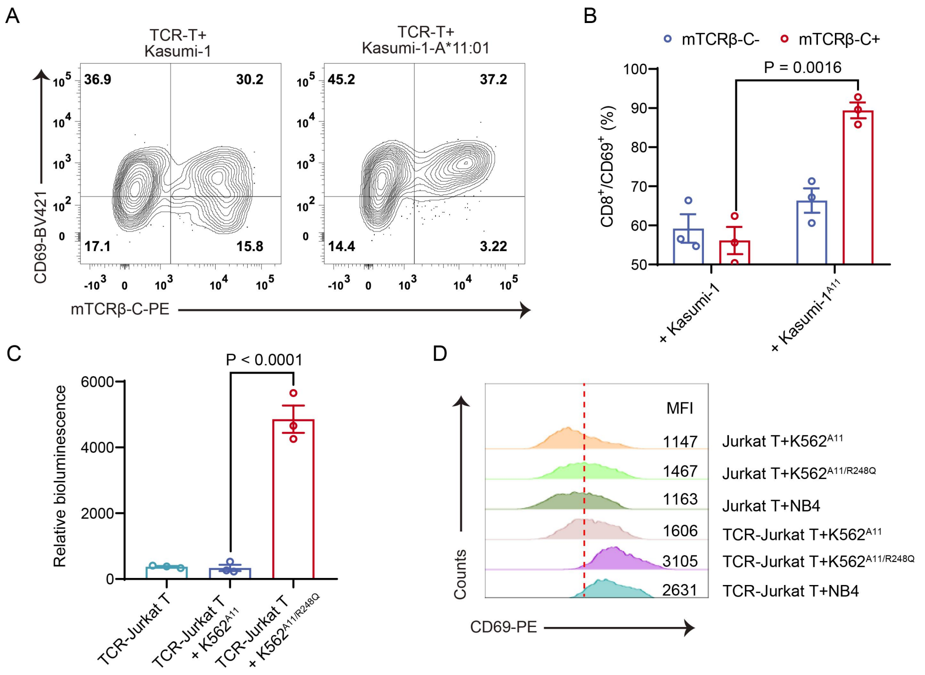

Supplemental Figure 3. In vitro specific activation assay of TP53^{R248Q} TCR-T. (A-B) Flow cytometry was performed to detect the expression levels of TP53^{R248Q} TCR-T activation marker CD69 after co-culture with Kasumi-1^{A11} cells or Kasumi-1 control cells (*n*=3). **(C)** The Jurkat T-NFAT-Luciferase reporter system was used to detect the level of TP53^{R248Q} TCR-T activation after co-culture with K562^{A11} control cell or K562$^{A11/R248Q}$ target cells (*n*=3). **(D)** After 24 hours of co-culture of jurkat T or TCR-jurkat T with K562^{A11} control cell or K562$^{A11/R248Q}$, NB4 target cells, MFI for CD69 was measured using flow cytometry to assess TCR-T activation. Data are presented as the mean ± SEM, *n*=3. In B, Two-way ANOVA was used. In C, One-way ANOVA was used.

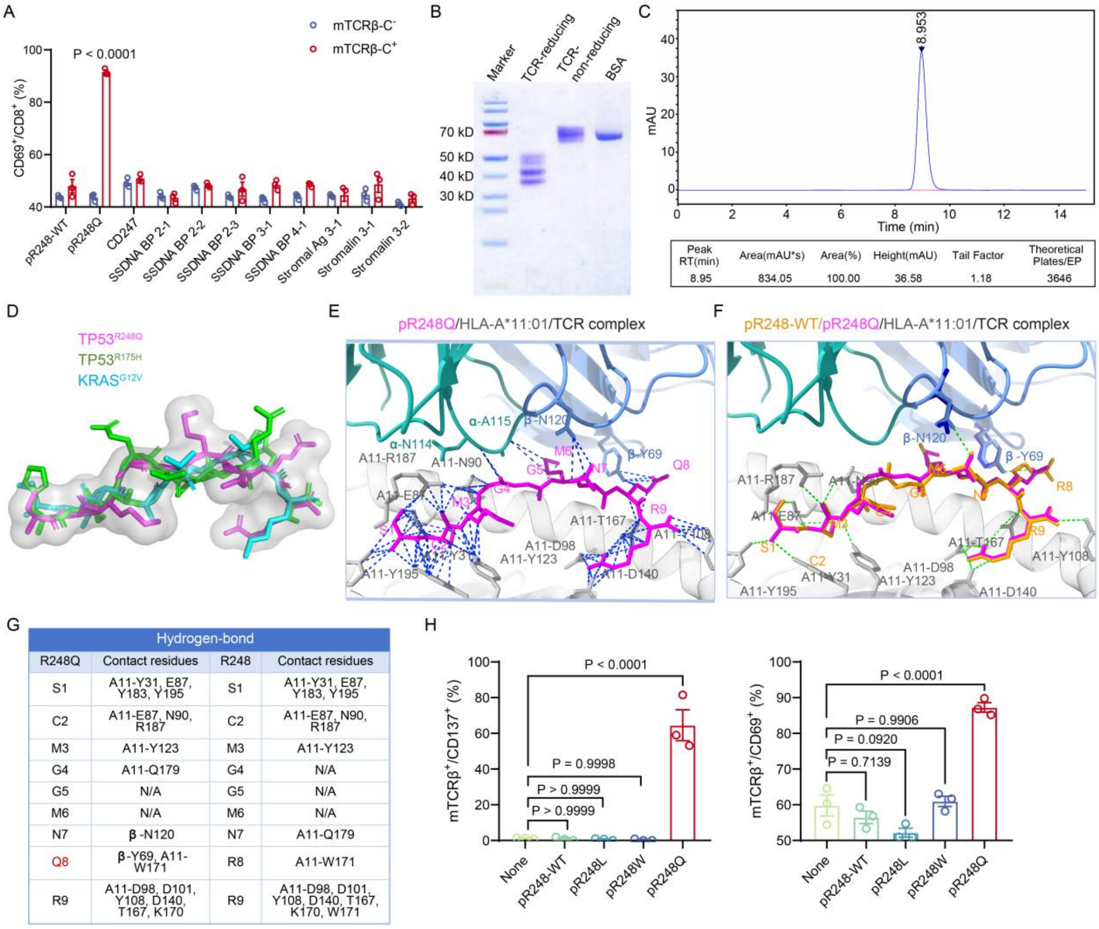

Hydrogen-bond			
R248Q	Contact residues	R248	Contact residues
S1	A11-Y31, E87, Y183, Y195	S1	A11-Y31, E87, Y183, Y195
C2	A11-E87, N90, R187	C2	A11-E87, N90, R187
M3	A11-Y123	M3	A11-Y123
G4	A11-Q179	G4	N/A
G5	N/A	G5	N/A
M6	N/A	M6	N/A
N7	β -N120	N7	A11-Q179
Q8	β-Y69, A11-W171	R8	A11-W171
R9	A11-D98, D101, Y108, D140, T167, K170	R9	A11-D98, D101, Y108, D140, T167, K170, W171

Supplemental Figure 4. Structural basis of pMHC recognition by TP53^{R248Q} TCR-T and detection of its recognition peptide specificity. (A) Nine predicted peptides were synthesized and loaded onto K562^{A11} cells, and then flow cytometry was performed to detect the expression levels of CD69 in TCR-T cells (*n*=3). **(B)** Bis-Tris Page was performed to analyze TP53^{R248Q} TCR at reducing and nonreducing condition respectively. **(C)** The recombinant TP53^{R248Q} TCR was analyzed by SEC-HPLC to evaluate its aggregation state and molecular integrity. **(D)** Structural homology analysis of TP53^{R248Q} Neoantigen reveals backbone conformational similarity with TP53^{R175H} and KRAS^{G12V} mutant epitopes. **(E)** Predicted possible binding of pR248Q to HLA-A*11:01 or TP53^{R248Q} TCR in the pR248Q/HLA-A*11:01/TCR complex. **(F)** Comparison of possible binding of

$TP53^{R248Q}$ or $TP53^{R248WT}$ peptides to HLA-A*11:01 or $TP53^{R248Q}$ TCR by Alphafold3 structure prediction. **(G)** Statistics on the binding of $TP53^{R248Q}$ mutant peptides or $TP53^{R248WT}$ peptides to HLA-A*11:01 or $TP53^{R248Q}$ TCR and their possible sites of hydrogen bond formation. **(H)** TCR-T activation was assessed by measuring CD69 and CD137 expression using flow cytometry after co-culturing TCR-T with $K562^{A11}$ control cells or $K562^{A11}$ cells loaded with the $TP53^{R248Q}$ neo-peptide and other mutations at position R248 for 24 hours. Data are presented as the mean ± SEM, *n*=3. In A, Two-way ANOVA was used. In H, One-way ANOVA was used. The p-values for **A** were relative to the $TP53^{R248WT}$ peptide group.

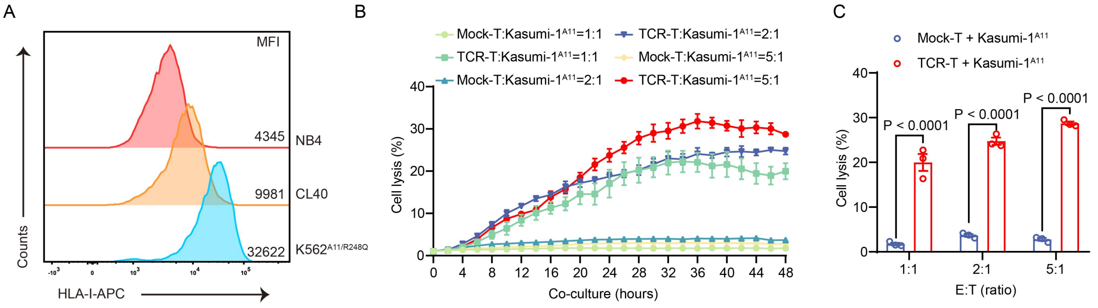

D

Pt. No.	$TP53^{R248Q}$ mutation	$TP53^{R248Q}$ rate (ddPCR)	HLA-A*11:01
1	-	N/A	+
2	+	8.79%	-
3	+	65.98%	+

E

Cell name	Cell type
$CD3^{+}$ T cell	T cells
$CD19^{+}$ B cell	B cells
$CD14^{+}$ Monocytes	Monocyte
HL-7702[L-02]-A *11:01	Human normal hepatocytes
MRC-5-A*11:01	Human normal embryonic lung cells
AC16-A*11:01	Human normal cardiomyocytes
BEAS-2B -A*11:01	Human normal lung epithelial cells
293T-A*11:01	Human normal embryonic kidney cells

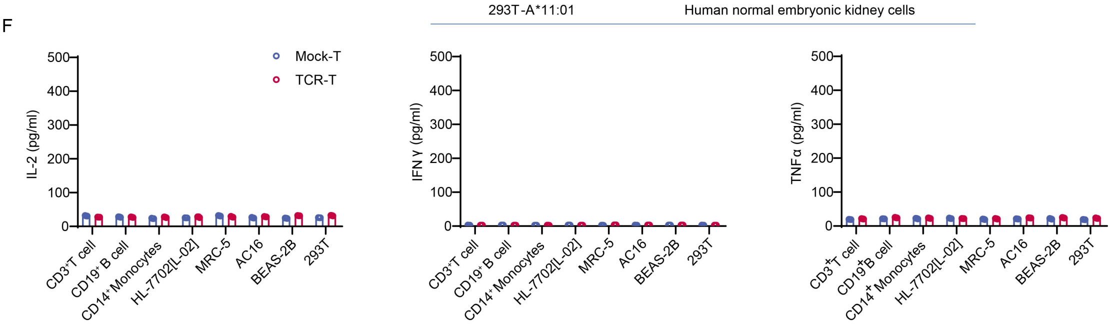

Supplemental Figure 5. Validate the killing efficacy and safety profile of $TP53^{R248Q}$ TCR-T cells in vitro. (A) Analysis of HLA-I expression level in different target cell lines. **(B-C)** Realtimie cytotoxicity of TCR-T Cells toward Kasumi-1^{A11} tumor cells at varying E:T ratios within 48 hours by Incucyte detect. n=3. **(D)** Clinical information of primary ALL patients: HLA-A*11:01 (HLA typing) and $TP53^{R248Q}$ status (ddPCR sequencing). **(E)** Information of primary human cells (T cell, B cell and monocytes) and part of human normal tissue-specific cell lines. **(F)** Analysis of cytokine secretion profiles (IL-2/IFN-γ/TNF-α) by ELISA revealed no statistically significant elevation of cytokine secretion profiles in TCR-T cells compared to the Mock-T control group. Data are presented as the mean ± SEM, n=3. In C and F, Two-way ANOVA was used.

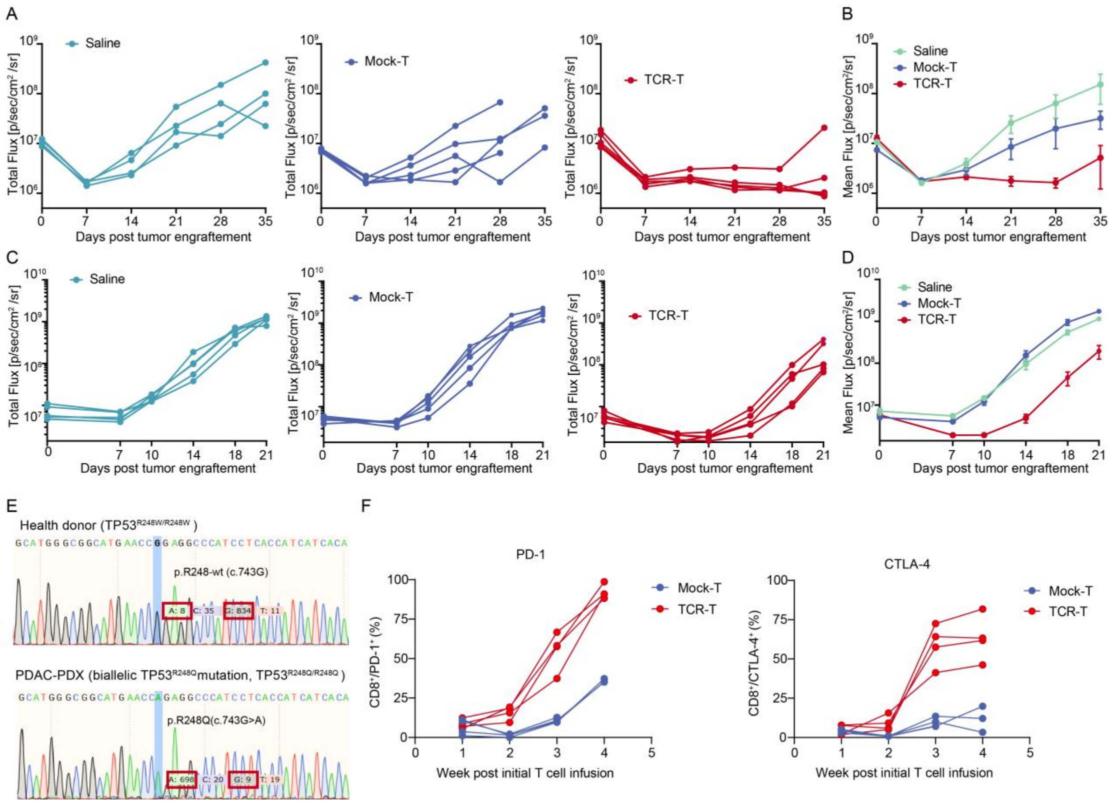

Supplemental Figure 6. In vivo anti-tumor efficacy detection of TP53^{R248Q} TCR-T cells. (A-B) In vivo killing efficacy assessment of TP53^{R248Q} TCR-T cells targeting K562$^{A11/R248Q}$ luciferase tumor cells. Total flux intensity of individual groups (each line represents a mouse), or comparison of mean flux intensity between groups. Saline group, *n*=4; Mock-T group, *n*=5; and TCR-T group, *n*=5. **(C-D)** In vivo killing efficacy assessment of TP53^{R248Q} TCR-T cells targeting NB4 luciferase tumor cells. Total flux intensity of individual groups (*n*=5), or comparison of mean Flux intensity between groups. **(E)** Gene sequencing to detect mutations at the TP53^{R248} locus in health donor or PDAC patients. **(F)** Dynamic changes in T-Cell exhaustion markers (PD-1 and CTLA-4) by flow cytometry, each line represents a mouse (*n*=4). Data are presented as the mean ± SEM.
